# Effect of isolated intracranial hypertension on cerebral perfusion within the phase of primary disturbances after subarachnoid hemorrhage in rats

**DOI:** 10.3389/fncel.2023.1115385

**Published:** 2023-07-12

**Authors:** Guangshan Hao, Catharina Conzen-Dilger, Tobias Philip Schmidt, Ekaterina Harder, Malte Schöps, Johanna Charlotte Clauser, Gerrit Alexander Schubert, Ute Lindauer

**Affiliations:** ^1^Translational Neurosurgery and Neurobiology, Department of Neurosurgery, Medical Faculty, RWTH Aachen University, Aachen, Germany; ^2^Department of Neurosurgery, Liaocheng People’s Hospital, Liaocheng, Shandong, China; ^3^Department of Neurosurgery, Medical Faculty, RWTH Aachen University, Aachen, Germany; ^4^Department of Cardiovascular Engineering, Institute of Applied Medical Engineering, University Hospital RWTH Aachen, Aachen, Germany; ^5^Department of Neurosurgery, Kantonsspital Aarau, Aarau, Switzerland

**Keywords:** subarachnoid hemorrhage, intracranial hypertension, inflammation, early brain injury, cerebral blood flow, cerebral autoregulation, animal model, rat

## Abstract

**Introduction:**

Elevated intracranial pressure (ICP) and blood components are the main trigger factors starting the complex pathophysiological cascade following subarachnoid hemorrhage (SAH). It is not clear whether they independently contribute to tissue damage or whether their impact cannot be differentiated from each other. We here aimed to establish a rat intracranial hypertension model that allows distinguishing the effects of these two factors and investigating the relationship between elevated ICP and hypoperfusion very early after SAH.

**Methods:**

Blood or four different types of fluids [gelofusine, silicone oil, artificial cerebrospinal fluid (aCSF), aCSF plus xanthan (CX)] were injected into the cisterna magna in anesthetized rats, respectively. Arterial blood pressure, ICP and cerebral blood flow (CBF) were continuously measured up to 6 h after injection. Enzyme-linked immunosorbent assays were performed to measure the pro-inflammatory cytokines interleukin 6 (IL-6) and tumor necrosis factor α (TNF-α) in brain cortex and peripheral blood.

**Results:**

Silicone oil injection caused deaths of almost all animals. Compared to blood, gelofusine resulted in lower peak ICP and lower plateau phase. Artificial CSF reached a comparable ICP peak value but failed to reach the ICP plateau of blood injection. Injection of CX with comparable viscosity as blood reproduced the ICP course of the blood injection group. Compared with the CBF course after blood injection, CX induced a comparable early global ischemia within the first minutes which was followed by a prompt return to baseline level with no further hypoperfusion despite an equal ICP course. The inflammatory response within the tissue did not differ between blood or blood-substitute injection. The systemic inflammation was significantly more pronounced in the CX injection group compared with the other fluids including blood.

**Discussion:**

By cisterna magna injection of blood substitution fluids, we established a subarachnoid space occupying rat model that exactly mimicked the course of ICP in the first 6 h following blood injection. Fluids lacking blood components did not induce the typical prolonged hypoperfusion occurring after blood-injection in this very early phase. Our study strongly suggests that blood components rather than elevated ICP play an important role for early hypoperfusion events in SAH.

## 1. Introduction

Aneurysmal subarachnoid hemorrhage (SAH), a devastating medical emergency with high mortality and morbidity, accounts for only 7% of all strokes but occurs in much younger patients compared with ischemic stroke (Feigin et al., 2003; Abraham and Chang, 2016). Despite advances in diagnosis and treatment, unfavorable prognosis, especially lifelong disability, has not improved substantially and thus still imposes a heavy burden on individual patients and the society (le Roux and Wallace, 2010). To further explore the pathophysiological mechanisms responsible for morphological and functional deficits in aneurysmal SAH, research interest has been shifted from delayed cerebral vasospasm to early brain injury (EBI). EBI refers to the multifaceted events during the first 72 h after the onset of SAH with the detailed pathophysiological processes still poorly understood (Cahill et al., 2006; Chowdhury et al., 2013; Fujii et al., 2013). Elevated intracranial pressure (ICP) and the extravascular distributed blood and its degradation products are the two important early trigger factors, which are discussed to mainly contribute to the initiation of EBI and the subsequently occurring cascade reactions (Bederson et al., 1995; Petzold et al., 2003; Naraoka et al., 2019). However, their individual role for the early impact on vascular and cellular function as well as for the development of further sequelae is not fully resolved so far. Initiated by a phase of primary disturbances preceding EBI, EBI thus represents the process of early secondary brain injury lasting up to 72 h in humans or up to 24 h in rodents (Fujii et al., 2013). According to a very recent review by Lauzier et al. (2023), we here define this very early phase up to 6 h after the onset of bleeding, focussed on by the present study, as primary disturbance phase (PDP) to better separate it from the subsequent EBI period. Aiming at a better understanding of the respective impact of the two main trigger factors within PDP on the further development of the pathophysiological cascade is not only a matter of basic-research interest, but may also be relevant from a clinical standpoint, because these two factors require different treatment strategies.

A suitable animal model for intracranial hypertension plays an important part in distinguishing effects between elevated ICP and blood components on brain damage in the context of various intracranial hemorrhagic diseases. For preclinical research of epidural, subdural or intraparenchymal hemorrhage, respectively, epidural placement of an inflatable balloon as well as subdural or intraparenchymal injection of an inert fluid (silicone oil or paraffin oil) have been successfully performed to develop intracranial hypertension models without any impact of blood components (Burger et al., 2002; Baechli et al., 2010; Krenzlin et al., 2020). However, after the onset of SAH blood distributes broadly within the subarachnoid space resulting in a unique ICP change pattern followed by more complex pathophysiological sequela. Following SAH, ICP elevates suddenly to a fairly high level and then decreases quickly to a much lower level, while remaining still significantly higher than baseline (Friedrich et al., 2013; Backer-Grondahl et al., 2016; Lu et al., 2017). To our knowledge, there is still no suitable animal model exactly reproducing the early course of intracranial hypertension during SAH that can be deployed to separately investigate the effects of elevated ICP and blood components.

Currently, two main animal models have been widely used for preclinical research of SAH, namely the endovascular perforation model and the blood injection model. Due to the inclusion of the effect of vascular damage during perforation, the former model may better represent human aSAH, however, the isolated impact of ICP changes cannot be investigated in this model (Buhler et al., 2015; Leclerc et al., 2018). As for the latter one, usually, identical volumes of 0.9% saline or artificial cerebrospinal fluid (aCSF) are typically injected as a control group, however, resulting in a much lower level of peak-ICP followed by quick return to baseline and thus not resembling the course of ICP following blood injection (Kolar et al., 2017; Conzen et al., 2019). Saline or aCSF, as pure crystal fluids, miss important blood-related characteristics: colloid osmotic pressure, structure-viscous behavior (shear-thinning), macromolecular substances, and cellular components. These characteristics are also responsible for the impaired CSF circulation following SAH, representing a critical parameter for progression of intracranial hypertension (Bothwell et al., 2019). Therefore, with this study we firstly aimed at modifying the typically applied aCSF based injection fluid to achieve an ICP course comparable with that occurring by blood injection. Secondly, we investigated the impact of the isolated, blood-injection-matched ICP-course on cerebral blood perfusion, autoregulation and early inflammatory tissue markers (Dumont et al., 2003; Geraghty et al., 2019) within the first 6 h after fluid injection using the well characterized cisterna magna injection model.

## 2. Materials and methods

### 2.1. Animals

Male Wistar rats (Janvier Labs, France), weighing 285–340 g, were used for the present study. Before surgery, all rats, housed in specific pathogen free and environmentally controlled room (22 ± 2°C, 55 ± 5% humidity and 12:12 h light/dark circle), could get access to food and water *ad libitum* and were given no less than 1 week to adjust to the new surroundings. All animal experiments were approved by the responsible state authorities in line with the EU Directive 2010/63/EU [Landesamt für Natur, Umwelt und Verbraucherschutz (LANUV) Nordrhein–Westfalen, Recklinghausen, Germany; AZ 84-02.04.2015.A412] and were conducted in accordance with the ARRIVE Guidelines.

### 2.2. Experimental design

#### 2.2.1. Experimental study 1

To establish a rat SAH-related intracranial hypertension model, 70 rats were allocated to 5 different groups with a monitoring period of 6 h after injection: SA-B: injection of autologous blood (*n* = 16), SA-G: injection of gelofusine (*n* = 16), SA-S: injection of silicone oil (*n* = 6), SA-C: injection of aCSF (*n* = 16) and SA-CX: injection of aCSF supplemented with xanthan (CX; final concentration of xanthan: 0.1%) (*n* = 16). Whereas for gelofusine, identical parameters for volume (0.5 ml) and injection time (1 min) as for blood injection were chosen, for the other fluid substitutes the parameters were varied in the attempt to best reproduce the ICP course of blood. In SA-C group, 1.5 ml aCSF was injected into cisterna magna within the first minute followed by continuous infusion at a rate of 2 ml/h till the end of the experiment. In SA-CX group, 1.0–1.2 ml CX was injected within the first minute followed by continuous infusion during the next 3 h with the rate adjusted according to the individual ICP course (range: 0–1 ml/h during first 30 min followed by 0–0.1 ml/h thereafter). Further information on the respective fluid composition and injection is provided in the Supplementary material. Brain tissue (basal and parietal cortex), and blood samples were collected at the end of the experiment. Due to organizational reasons in order to find and establish a suitable fluid composition for injection, the surgeon was not blinded to fluid type. The experiments were conducted subsequently with SA-CX performed as the last study group, thus randomization was not possible.

#### 2.2.2. Experimental study 2

To investigate the very early inflammatory response within the first 2 h following blood or CX injection, 12 rats were allocated to 2 different groups, in which the same amount of fluid as in experimental study 1 was injected. In this project, blood samples were withdrawn at baseline and every 30 min after the start of the injection. Brain tissue was collected at the end of the experiment. The experiments were performed in a randomized way, with blinding of the surgeon not possible due to visual identification of the fluid (blood or CX, respectively). Detailed information is provided in the Supplementary material.

#### 2.2.3. Estimation of sample size:

For Experimental study 1, sample size for each group was calculated by *a priori* power analysis (free software G*Power 3.1.7; power: 1-β = 0.8; α = 0.05) using data for CBF, ICP, ABP and CA from former experiments from our group using a similar study design. A maximum of *n* = 16 per group was calculated, with potential dropouts included in this number. For SA-S, an identical number was originally planned, however, after conduction of six experiments with spontaneous death of all except one animal right after fluid injection, the work on this group was prematurely terminated due to ethical reasons.

For experimental study 2, the number of animals was determined by experience from former studies for assessment of inflammatory mediators in blood and tissue, with no *a priori* power calculation performed.

### 2.3. Viscosity measurement

A tempered cone plate rheometer (Anton Paar MCR502 Cone: CP60–1/TG with Plate: C-PTD200; Anton Paar GmbH, Graz, Austria) was used to measure viscosity of blood and all blood substitutes included in the present study. Measurement was repeated three times for each solution. In the range of logarithmic subdivisions 5 s^–1^ to 2,000 s^–1^, dynamic viscosity of each sample was recorded at 22 measuring points. Parameters: cones’ diameter 59.983 mm, angle 0.981°, gap size 0.121 mm, T 37°C. Sample volume for measurement: 0.974 ml.

### 2.4. Surgery

Based on our well-established blood injection model with 0.5 ml autologous arterial blood injected into cisterna magna within 1 min, all surgery procedures were performed as previously described (Conzen et al., 2019). A more detailed description is provided in the Supplementary material. Briefly, appropriate anesthesia and analgesia [isoflurane (1.6–2%) combined with intravenous fentanyl infusion (0.015 mg/kg/h), complemented by subcutaneous ropivacaine application at each skin incision] was frequently checked and verified by the lack of response to toe pinch. Following tracheotomy, the animals were mechanically ventilated, and femoral artery and vein was catheterized. Body temperature, arterial blood pressure (ABP) and arterial blood gases were monitored and kept within the physiological range. After fixation of the rat’s head in a stereotactic frame, a cranial window (bone intact while thinned to translucency) was implanted over the right parietal cortex to measure changes of local cerebral blood flow (CBF). Electroencephalogram (EEG), not further analyzed in the present study, was monitored with an epidurally placed electrode. A microcatheter, placed into the cisterna magna, was used to inject blood or different blood substitutes as planned. The injection was performed by a small volume syringe pump. ICP was measured via a microcatheter inserted into the subarachnoid space through the dorsal atlantooccipital membrane. Assessment of ICP at this location leads to comparable values as measured with a pressure sensor placed epidurally within the parietal cortex (data from our own group, not published).

At the end of the recording period, animals were euthanized in deep anesthesia by an intravenous overdose of 2 ml 2.5 M potassium chloride. After decapitation, the brain was removed and rinsed using saline. High-resolution photographs were taken to verify and document successful SAH in SA-B or absence of subarachnoid blood in all other groups, respectively.

### 2.5. Monitoring

**FIGURE 1:**
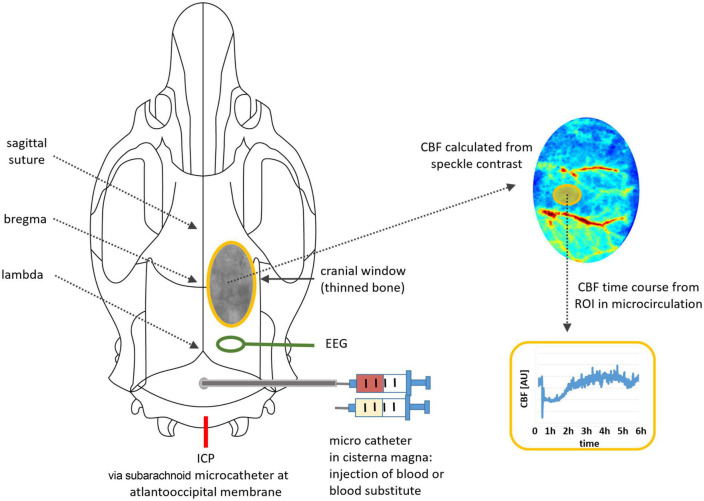
Illustration of the experimental setup: positions of microcatheters for fluid injection, for ICP monitoring and for EEG electrode, cranial window with thinned bone, image processing for time course analysis.

The rat was transferred to a platform for CBF monitoring by laser speckle contrast analysis [Superficial Tissue Imaging System (STIS), Biomedical Optics Laboratory, RheinAhrCampus Remagen, Germany]. ICP, ABP and EEG were continuously monitored via a data acquisition interface (power 1401, Spike2 8.02, Science Products, Hofheim, Germany). CBF, ICP, ABP and EEG were continuously monitored with recording started 30 min before injection and lasting up to 6 h after fluid injection, as planned. A schematic overview of the experimental setup is provided in Figure 1.

### 2.6. Data processing

Raw data of ICP, ABP and CBF was processed and cerebral perfusion pressure (CPP) and index of cerebral autoregulation were calculated as previously described (Bach et al., 2019; Conzen et al., 2019), with the investigator blinded to group allocation.

Briefly, ICP and ABP courses were analyzed with frequency set at 1 Hz using spike software (Cambridge Electronic Design, Cambridge, GB). CPP was calculated as the difference between ABP and ICP.

For assessment of cerebral autoregulation (CA), the pressure reactivity index (PRx), one commonly used index for CA, was calculated as the continuous Pearson correlation coefficient between ICP and ABP at low frequencies, as previously reported (Czosnyka et al., 1997; Conzen et al., 2019). Baseline level was obtained by averaging 20 consecutive PRx values for a period of 60 s. After the start of injection, index values per minute were calculated as an average of 30 consecutive PRx values of each 60 s. A positive PRx > 0.2 indicates impaired CA, whereas PRx < 0.2 reflects an intact CA. According to their respective PRx curves, each animal in each group was allocated to the following categories according to the %-age of datapoints above or below the cutoff value of 0.2: category mostly impaired CA: more than 50% of the datapoints are > 0.2; category transiently impaired CA: less than 50% but more than 0% of all datapoints are > 0.2; category intact CA: 0% of the datapoints are > 0.2 (= all datapoints are < 0.2).

For CBF assessment, raw speckle pattern images were converted online into contrast images by STIS (recorded at 0.5 Hz frequency) and then offline into images of CBF changes, applying a self-written MATLAB script [provided by Steimers et al. (2013) Biomedical Optics Laboratory, RheinAhrCampus Remagen, Germany; MATLAB software: version 7.3.0.267/R2006b, The MathWorks, USA]. To analyze CBF time courses, regions of interest were located within the microcirculation. Data of relative CBF was presented as percentage change from baseline which was calculated as the average CBF value within 5 min prior to fluid injection and set at 100%. For statistical analysis, baseline CBF was presented as the average of the data 1 min prior to fluid injection.

Artifacts within recorded data (for example in ABP induced by clamp of arterial catheter for blood sampling; in CBF unusual short high peaks induced by mechanical disturbance) were removed and replaced by mean value data points calculated from data directly before and after the artifact before further processing.

### 2.7. Enzyme-linked immunosorbent assay

Arterial blood samples were withdrawn at different time points as designed. Plasma samples were then obtained after centrifugation (1,000 rcf, 20 min, 20°C) and stored at −80°C. Parietal cortex and basal cortex samples of each left hemisphere were collected as previously described (Bach et al., 2019). Briefly, after adding RIPA buffer, tissue samples were homogenized using a shredder column (QIA Shredder; Qiagen Inc., Hilden; Germany). Supernatant was collected after centrifugation (4,000 rpm, 2 min at 4°C) and stored at −80°C until measurement.

IL-6 and TNF-α concentrations in plasma and brain cortex tissues were measured using commercial enzyme-linked immunosorbent assay (ELISA) kits (Plasma samples: R6000B for IL-6, RTA00 for TNF-α, R&D, Minneapolis, USA; Brain cortex samples: ERA32RB for IL-6, ERA57RB for TNF-α, ThermoFisher, Carlsbad, USA) in line with manufacturer’s manual. At the end, following the addition of stop solution to terminate the reaction, optical density was immediately analyzed at 450 nm using a microplate reader (Synergy HT Multi-Mode Microplate Reader; BioTek, Winooski, USA) with a second reading at 540 nm for wavelength correction.

### 2.8. Statistical analysis

GraphPad Prism 9 (GraphPad, San Diego, CA, USA) was used as a statistical analysis tool for the present study. Shapiro-Wilk normality test was applied to test for normal distribution, showing that for each parameter the data at specific time points did not pass the test. Significance testing was yet performed by ANOVA since this analysis is commonly regarded as robust against violation of the requirements of normal distribution as well as equal variance (with equal sample size). Two-way ANOVA followed by Dunnett’s or Šídák’s multiple comparisons test was applied to test for differences between groups and deviations from baseline within groups, respectively. Ordinary one-way ANOVA followed by Dunnett’s or Šídák’s multiple comparisons test was used to analyze differences in inflammatory response of tissue samples at 2 or 6-h time point or of plasma samples at 6-h time point, respectively. Due to missing values, for ABP and CPP, mixed-effects model (REML) followed by Dunnett’s multiple comparisons test was applied to test for differences between groups and deviations from baseline within groups. Statistical significance was accepted when *p*-value was less than 0.05. Within the text, quantitative values are presented as median (75% percentile; 25% percentile). The box plot figures show boxes with median and 25 and 75% percentiles and whiskers representing minimum and maximum values, respectively. The time course figures show mean values only.

## 3. Results

### 3.1. General information

**FIGURE 2:**
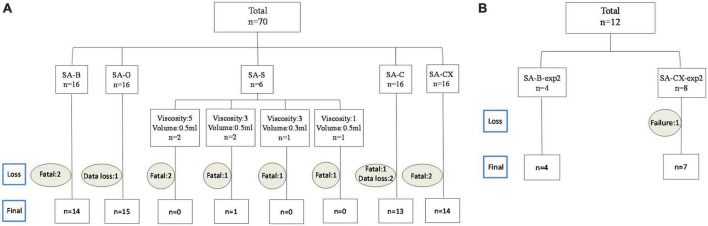
Overview on experimental group allocation and drop out numbers: **(A)** experimental study 1; **(B)** experimental study 2.

In Experimental study 1, individuals with missing data (*n* = 3: one from SA-G group and two from SA-C group) or individuals that died after injection before reaching the planned recording endpoint (*n* = 10: two from SA-B group, five from SA-S group, one from SA-C group and two from SA-CX group) were excluded from the analysis (Figure 2A). In experimental study 2, one in SA-CX group was also excluded due to the failure of ICP monitoring (Figure 2B). Blood was found covering basal cistern or parietal and temporal surface with arteries visible or invisible in SA-B group and was absent in all other groups (Supplementary Figure 1). Parameters of blood gas analysis were maintained in normal range throughout the experiments (data not shown).

### 3.2. Viscosity levels of fluids used in the experiments

**FIGURE 3:**
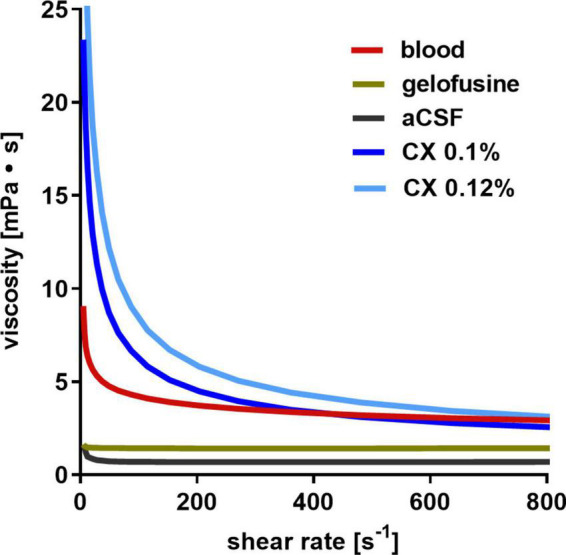
Dynamic viscosity of fluids: whereas aCSF and gelofusine show a low dynamic viscosity, aCSF with 0.1% xanthan (CX) best resembles that of blood.

The measured relationship between viscosity and shear rate for the fluids is depicted in Figure 3. The exact shear rate of fluid in the subarachnoid and perivascular space is not known and hard to calculate due to its irregular morphological characteristics. Considering the comparably low flow rate of CSF within the subarachnoid space, a shear rate in the lower range can be assumed. The measured viscosity of gelofusine and aCSF was almost constant at different shear rates and considerably lower than that of blood, supposing that they may be less suitable for ICP modulation. Raising the viscosity of aCSF by addition of 0.1% xanthan (CX 0.1%) resulted in fluid characteristics that reasonably resembled that of blood, with this fluid composition therefore used in the SA-CX group.

### 3.3. Silicone oil injection caused rapid death

After not succeeding our goal of modulating the blood-injection induced ICP course with gelofusine at identical volume as blood, we decided to use silicon oil that is frequently used to mimic subdural or intraparenchymal hemorrhage. We successively tried several silicone oil types of different viscosity levels and injection volumes: (1) viscosity 5, 0.5 ml injected within 1 min (*n* = 2); (2) viscosity 3, 0.5 ml injected within 1 min (*n* = 2); (3) viscosity 3, 0.3 ml injected within 1 min (*n* = 1); (4) viscosity 1, 0.5 ml injected within 1 min (*n* = 1). Five of these six rats died after injection. Thus, for ethical reasons and experimental principles, this study group was prematurely terminated, and no more rats were enrolled in this group. Separate from a figure depicting the ICP course within the first minutes, showing a smaller peak compared with blood injection (Supplementary Figure 2), a detailed analysis of the recorded data up to the death of the respective animal was not performed.

### 3.4. ICP: CX injection best resembled ICP course of blood injection

**FIGURE 4:**
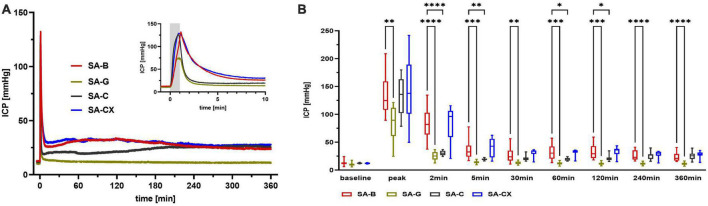
ICP: **(A)** ICP courses induced by the fluids throughout the measurement of 6 h, insert: first 10 min enlarged (gray area depicting the 1 min fluid injection period). **(B)** Quantitative analysis at distinct time points (****p < 0.0001; ***p < 0.001; **p < 0.01; *p < 0.05).

While analyzing the time courses of ICP, in all groups an instant and remarkable ICP elevation occurred during the first minute of fluid injection with maximum values (peak) reached at the end of the one-minute injection time (Figure 4A). In comparison with SA-B, SA-G, with the same amount of fluid injected as in SA-B, caused a significantly smaller ICP elevation, whereas both aCSF (SA-C) and aCSF + xanthan (SA-CX), with higher volumes applied, injection induced comparable ICP peak values following the 1 min of injection. During the further course, ICP rapidly decreased in all groups, with injection of SA-B showing the well-known elevated plateau until the end of the recording period. In SA-G, ICP remained significantly elevated only up to the 5 min time point and returned back to baseline values within 30 min after injection, with significantly lower values compared with SA-B for all time points. In SA-C, due to increased bolus volume injection followed by high continuous infusion rate, ICP remained significantly heightened compared with baseline throughout the recoding period. However, compared with SA-B, ICP in SA-C remained lower up to 120 min after start of injection. In SA-CX group, in which aCSF viscosity level was increased by xanthan, the injection volume and rate were decreased in respect to SA-C and adjusted during the course of the experiment to achieve a comparable distribution of ICP time courses as in SA-B. As a result, SA-CX showed an almost identical ICP time course as SA-B group throughout the experiment (Figure 4B, Supplementary Figure 3, and Supplementary Table 1).

### 3.5. ABP and CPP

After start of fluid injection, time courses of ABP showed the well-known, ICP-induced instantaneous transient increase with return to baseline values within 5–10 min (Cushing reflex) in all groups with significantly lower reaction in SA-G (Supplementary Figure 4 and Supplementary Table 3). SA-CX showed a second transient, albeit smaller, elevation peaking at 60 min.

Details on calculated courses of CPP are shown in the Supplementary material (Supplementary Figure 5 and Supplementary Table 4). Briefly, all groups showed a strong reduction of CPP within the first minute during the bolus injection of the fluids. Further on, SA-G resulted in even elevated CPP values within the first 10 min and showed preserved CPP during the further course of the experiments, with all time points being significantly higher compared with blood injection. In SA-B and SA-CX CPP was reduced throughout the recording period with no difference between these groups. In SA-C, the sharp CPP reduction during bolus injection was followed by a short transient CPP elevation within the first few minutes which was followed by a CPP reduction for the next 60 min with CPP recovery for the rest of the recoding period. Aside from the transient CPP elevation within the first minutes after the bolus, which did not occur in SA-B, CPP was comparable between SA-C and SA-B for the first hour. Later on, CPP was slightly but significantly higher in SA-C compared with SA-B for the next 2 h with an approximation of the courses for the remaining 3 h.

### 3.6. CBF: Elevated ICP without blood components causes early transient global ischemia with no further hypoperfusion

**FIGURE 5:**
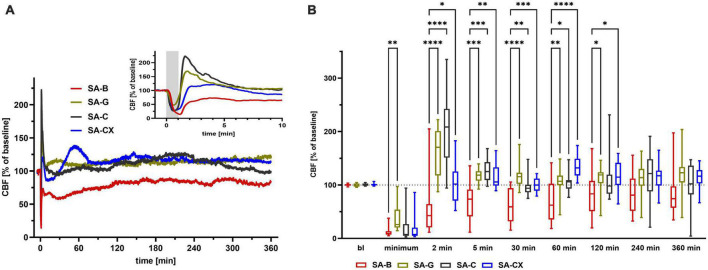
CBF: **(A)** CBF courses induced by the fluids throughout the measurement of 6 h, insert: first 10 min enlarged (gray area depicting the 1 min fluid injection period). **(B)** Quantitative analysis at distinct time points (****p < 0.0001; ***p < 0.001; **p < 0.01; *p < 0.05).

Along with the high ICP increase during the first minute with peak values at the end of the bolus injection, SA-C and SA-CX groups showed a comparable drop in CBF as SA-B group, whereas SA-G group showed a significantly smaller drop. After this early strong ischemia, CBF only partially recovered in SA-B with a significant hypoperfusion maintained for almost 2 h followed by slightly albeit not significantly reduced values until the end of the recording period. In all other groups, no hypoperfusion occurred after the short transient ischemia at the beginning. Instead, in SA-G and SA-C a reactive hyperemia developed for up to 5 min followed by a stable return to baseline CBF for the 6 h recording. In SA-CX, following the early strong ischemia, the transient hyperperfusion shown in SA-G and SA-C within the first minutes was missing, and apart from a delayed small hyperperfusion around 60 min, CBF remained unchanged from baseline. Compared with blood injection in SA-B, all other injected fluids (SA-G, SA-C and SA-CX) induced significantly higher CBF values up to 60 min (SA-C) or 120 min (SA-G, SA-CX) after injection, respectively. For the remaining 4 h, no difference in CBF was observed between all groups (Figure 5, Supplementary Figure 6, and Supplementary Table 2).

### 3.7. Cerebral autoregulation: PRx depicts impaired autoregulation only after blood injection

**FIGURE 6:**
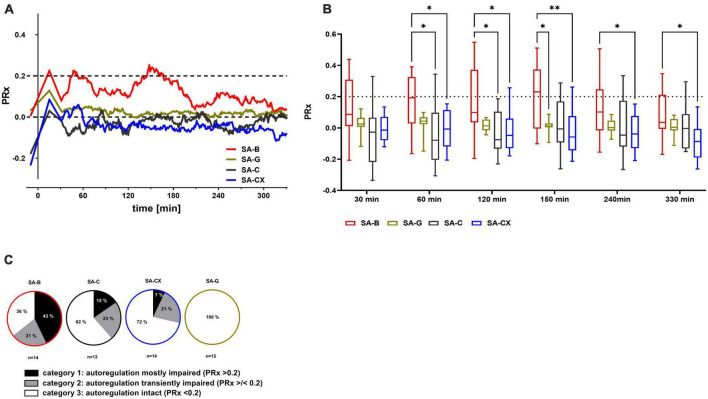
PRx as measure for cerebral autoregulation following fluid injection: the pressure reactivity index PRx, for assessment of cerebral autoregulation (CA) was calculated as the continuous Pearson correlation coefficient between intracranial pressure and arterial blood pressure at low frequencies. PRx > 0.2 indicates impaired CA, whereas PRx < 0.2 points toward an intact CA. **(A)** Median of PRx time courses for all groups. **(B)** Quantitative analysis at distinct time points (**p < 0.01; *p < 0.05). **(C)** Distribution between categories of mostly (more than 50% of the datapoints are > 0.2) or transiently (less than 50% but more than 0% of all datapoints are > 0.2) impaired or intact (0% of the datapoints are > 0.2, means all datapoints are < 0.2) CA according to the cutoff value of PRx 0.2 (% of animals in each group).

Cerebral autoregulation (CA) was intact at baseline in all groups. Following blood injection (SA-B), mean PRx values approximated 0.2 indicating a strong tendency toward impaired CA which lasted for about 160 min with a partial amelioration thereafter. Following fluid injection in all other groups (SA-G, SA-C, SA-CX), mean PRx remained clearly below 0.2 throughout the experiment pointing toward an intact CA (Figure 6A). Quantitative analysis of PRx at defined time points showed significantly higher values in SA-B compared with SA-C at 60 min and 120 min or with SA-CX at 60, 120, 150, 240 and 330 min (Figure 6B). While investigating the PRx courses of each single experiment in more detail, a subgrouping was performed by allocation of each animal’s dataset into one of the following categories: category 1: PRx > 0.2 most of the recording time; category 2: PRx undulant around 0.2; category 3: PRx < 0.2 all the time. In SA-B, with 64% the majority of the experiments belonged to category 1 and 2 (category 1: 43%, category 2: 21%) and thus at least transiently suffered from an impaired CA, whereas in 36% CA remained intact. In SA-C, only 38% of the experiments belonged to category 1 and 2 (category 1: 15%, category 2: 23%) whereas in 62% CA remained intact. In SA-CX, with 28% even fewer experiments belonged to category 1 and 2 (category 1: 7%, category 2: 21%) with a higher proportion showing intact CA (72%). In SA-G for all animals PRx remained < 0.2 throughout the monitoring phase (category 3: 100%) (Figure 6C, Supplementary Figure 7, and Supplementary Table 5).

### 3.8. Inflammatory response in blood and tissue following fluid injection

Pro-inflammatory cytokines (IL-6 and TNF-α), both in brain (basal cortex and parietal cortex) and plasma samples, were measured at 6 h after injection in experimental study 1 groups of SA-B, SA-C, and SA-CX, whereas in SA-G only basal cortex was analyzed while no parietal cortex and plasma samples were collected.

Regarding gelofusine (SA-G), aCSF (SA-C) or blood (SA-B) injection, there was no significant difference in plasma or tissue samples between these groups at 6 h after fluid injection for both cytokines, respectively.

**FIGURE 7:**
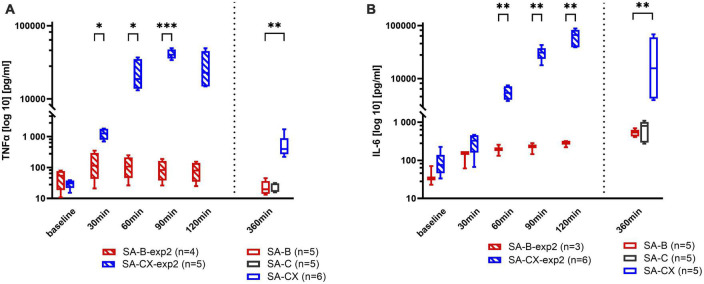
Systemic inflammatory response assessed in blood samples: **(A)** TNF-α, **(B)** IL-6. Data from experimental study 2, with samples taken every 30 min up to 2 h after blood or CX-injection (SA-B-exp2; SA-CX-exp2) are arranged together with data from experimental study 1, with samples taken at the end of the 6 h observation period (SA-B; SA-C; SA-CX) (***p < 0.001; **p < 0.01; *p < 0.05).

Following aCSF + xanthan injection (SA-CX), compared with blood injection, an unexpected strong systemic inflammatory reaction was depicted at 6 h with significantly elevated levels of IL-6 and TNF-α in blood samples (Figure 7 and Supplementary Tables 6, 7).

To reveal the early time course of this systemic inflammatory response, in experimental study 2, blood samples were taken at baseline and every 30 min after the start of the injection up to 2 h in SA-B-exp2 and SA-CX-exp2. The limit of up to 2 h was set as the time of the most prominent hypoperfusion of CBF in SA-B compared with SA-CX that completely lacked hypoperfusion at this time intervall. Again, aCSF + xanthan in SA-CX-exp2 induced a significantly stronger inflammatory response also at this early time interval than blood in SA-B-exp2. IL-6 reached a maximum level at the end of the 2-h period in experimental study 2 that was comparable with the concentration measured at 6 h in experimental study 1. TNF-α reached an early plateau response within the first hour in experimental study 2 with significantly higher values in SA-CX-exp2 compared to SA-B-exp2. At 6 h, TNF-α in SA-B and SA-C injection groups (experimental study 1) revealed concentrations comparable to the levels at baseline measured in experimental study 2, whereas following SA-CX injection, TNF-α was still significantly elevated compared with SA-B, however, at a notably lower level compared with the plateau response within the first 2 h in experimental study 2 (Figure 7).

**FIGURE 8:**
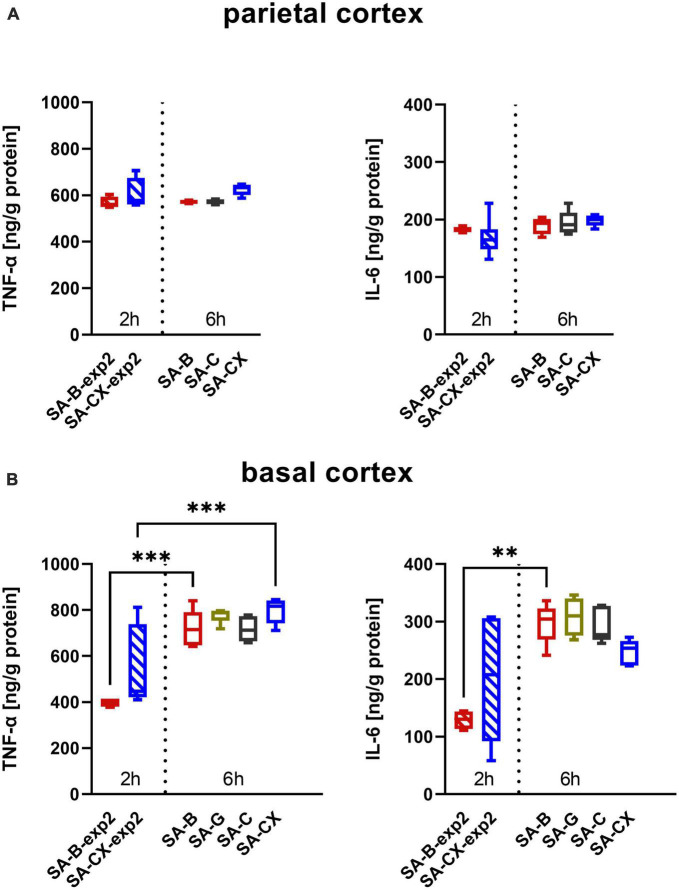
Inflammatory response from brain samples: **(A)** parietal and **(B)** basal cortex assessed by TNF-α (left) and IL-6 (right) analysis: data from experimental study 2, with samples taken at 2 h after blood or CX-injection (SA-B-exp2; SA-CX-exp2) are arranged together with data from experimental study 1, with samples taken at the end of the 6 h observation period (SA-B; SA-C; SA-CX) (***p < 0.001; **p < 0.01).

In tissue samples, levels of both cytokines IL-6 and TNF-α from animals of experimental study 1 at 6 h did not show any difference between the three (parietal cortex: SA-B, SA-C, SA-CX) or four groups (basal cortex: SA-B, SA-G, SA-C, SA-CX), respectively (Figure 8). Cytokine levels in tissue samples harvested at 2 h time point in experimental study 2 also did not show a significant difference between SA-B-exp2 and SA-CX-exp2. In addition, no concentration change of both cytokines was detectable in samples of the parietal cortex between 2 and 6 h following blood or aCSF + xanthan injection. In basal cortex, significantly larger concentrations of both cytokines were detectable after blood injection at 6 h (SA-B) compared with 2 h (SA-B-exp2) whereas following aCSF + xanthan injection, only TNF-α significantly increased between 2 and 6 h sampling time points (Figure 8 and Supplementary Tables 8, 9).

## 4. Discussion

In this study, we successfully designed a new rat intracranial hypertension model by injection of aCSF into the cisterna magna, supplemented with xanthan for increased viscosity (SA-CX). With a one-minute bolus followed by a small amount of continuous infusion it was possible to exactly reproduce the ICP time course of our standard blood injection model for subarachnoid hemorrhage in rats. Although CX-injection-induced ICP changes exactly followed that of blood injection, the CBF time course did not show the early hypoperfusion typically occurring after blood injection, pointing toward blood components rather than elevated ICP as the main contributor to early hypoperfusion in EBI following SAH. While injecting the other blood-substitution fluids with lower viscosity, aCSF or gelofusine, a comparably high ICP peak response within one minute was only achieved by high volume injection (SA-C), whereas it was not possible to reproduce the following blood-injection induced elevated ICP-plateau even with a high amount of continuous infusion. The early inflammatory response within the parietal and basal cortex did not differ between all injected fluids, rendering mainly the early ICP peak as the most important trigger for this early response. However, while injecting CX, the systemic inflammatory response was significantly higher and started very early compared with blood or the other fluids. A rapid flow of the non-corpuscular fluids along the ventricles and the perivascular glymphatic system may be suggested, with specific strong activation of inflammatory cells within the nasal or cervical lymphe nodes by xanthan. For overview, a summary of the main results is given in Table 1.

**TABLE 1:** Overview of results.

	Fatal/total	Peak ICP	Plateau ICP	Instant hypo-perfusion	Further hypo-perfusion	Neuro-inflammation	Systemic inflammation
SA-B	2/16	Yes	Yes	Yes	Yes	Yes	Yes**
SA-G	0/16	Yes*	No	Yes*	No	Yes	–
SA-S	5/6	Yes	–	–	–	–	–
SA-C	1/16	Yes	Yes*	Yes	No	Yes	Yes**
SA-CX	2/16	Yes	Yes	Yes	No	Yes	Yes

Yes*, significantly different (ICP: smaller; hypoperfusion: CBF larger) compared with SA-B. Yes**, significantly smaller compared with SA-CX.

Blood breaking into the subarachnoid space after aneurysm rupture and the accompanying ICP elevation are considered as the two main trigger factors for the following pathophysiological cascade of secondary EBI (Bederson et al., 1995; Petzold et al., 2003; Naraoka et al., 2019). However, it is not yet known whether they independently contribute to ischemia and hypoperfusion during PDP. To separate the contribution of both factors and investigate the isolated effect of elevated ICP after SAH, several procedures have been applied to modify existing SAH animal models. Siler et al. (2014) applied a CSF drainage system to decrease the elevated ICP starting 15 min after the induction of SAH in the endovascular perforation model in mice. However, it did not take into account the effect of the sudden ICP elevation during the first few minutes after the onset of SAH, and the CSF drainage system not only decreased elevated ICP but also partially washed out the blood components and thus reduced their potentially toxic effects. In another approach, while considering the beneficial effects of decompressive craniotomy (DC) for intracranial hypertension in clinical trials, prophylactic DC was performed before SAH in mice or rats to explore the effect of elevated ICP. However, a dramatically increased severity of bleeding and incidence of rebleeding was observed in the endovascular perforation model, and herniation induced increase in vascular resistance probably resulted in an even intensified hypoperfusion in the blood injection model in rats (Buhler et al., 2015; Kolar et al., 2017). In addition, in a rabbit blood-shunt model, in which arterial blood was directed into the subarachnoid space from a systemic artery through an extracorporeal shunt, subgroups with various levels of peak-ICP were achieved by stopping blood flow manually. However, blood volume flowing into subarachnoid space also varies significantly between the subgroups, rendering differentiation between effects of blood or ICP difficult (Andereggen et al., 2014; Marbacher et al., 2015; Lu et al., 2017). In a recent study from our group, blood infusion duration was prolonged in the rat blood injection model to achieve a reduced ICP peak (Conzen et al., 2019). All aforementioned studies were designed to reduce the effect of ICP while preserving the appearance of blood in the subarachnoid space, and thus only indirectly assessed the effect of intracranial hypertension compared with blood components.

In our effort to design an SAH-analog, isolated intracranial hypertension model, we used our well known SAH blood injection model as the basis. In the blood injection model, a good control over blood volume and time of pouring exists (Conzen et al., 2019), and blood can be replaced by other fluids to exclude blood-related effects. Considering the critical role of disturbed CSF circulation in intracranial hypertension due to restriction of fluid routes by blood after SAH, four different types of fluids with enhanced colloid osmotic pressure and/or viscosity compared with normal physiological saline were selected to replace blood. For the first try, gelofusine, a safely used plasma volume substitute in clinic which features the same colloid osmotic pressure as blood and has a higher viscosity level than saline or aCSF, was selected as a blood substitute, applying identical injection parameters of volume and rate as in the standard blood injection model. Following gelofusine injection, ICP achieved a higher peak-ICP level than saline injection group reported previously in our or others’ studies (Kolar et al., 2017; Conzen et al., 2019). Unfortunately, SA-G group still showed a significantly lower ICP course compared with SA-B group, not only at peak time but also during further 6-h monitoring time, which may be explained by the still low viscosity of gelofusine. Silicone oil or paraffin oil, an inert fluid, with a viscosity level of 5 has been successfully used to establish an intracranial hypertension model for intraparenchymal or subdural hemorrhage (Baechli et al., 2010; Krenzlin et al., 2020). However, even when trying silicone oil of lower viscosity and reducing the injection volume, most of the rats died in the present study immediately after the injection without developing dramatically high ICP elevation. Due to the hydrophobicity of silicone oil, acute obstructive hydrocephalus and brainstem compression might have been the lethal causes. Next, we tried to achieve an ideal ICP time course via continuous aCSF infusion exceeding the reabsorption rate which imitates an impaired CSF circulation event. Therefore, high amount bolus injection of 1.5 ml during the first minute, followed by continuous infusion at a rate of 2 ml/h, which is approximately 10 times the CSF exchange rate in rat, was used in SA-C group (Chiu et al., 2012; Murtha et al., 2014). In comparison with SA-G group, higher ICP levels both at peak-time and plateau-time were achieved in SA-C group. However, while identical peak values could be achieved, a significantly lower ICP plateau occurred during the first 2–3 h compared with blood-injection group. It can be speculated that due to the low viscosity, aCSF rapidly flows through the drainage pathways of the glymphatic system out of the brain with no accumulation and thus no relevant effect on ICP plateau. Further increasing the aCSF infusion amount was considered to be inappropriate, as hypertension induced pathophysiological vasoactive metabolites may be even more effectively washed out from the brain, thereby changing the perivascular environment and subsequently ameliorating any vascular impact. To reduce the necessary amount while retaining the tissue-inert properties of aCSF, we added xanthan to aCSF to increase its viscosity to the level of blood as a refinement for the next study. Finally, while continuously adjusting the infusion rate throughout the experiment, we were able to achieve almost identical ICP, ABP and CPP time courses in SA-CX group as in SA-B group. Simultaneously, the amount of fluid injection in SA-CX group could be significantly reduced compared with SA-C group, mitigating the possible protective impact of perivascular fluid flow and metabolite washout. Of note, despite CX featuring almost the same viscosity level as blood, both a bigger amount for bolus injection during the first minute and a small amount of continuous infusion during the first 3 h were needed in SA-CX group to achieve the same ICP course as SA-B group. Not only the viscosity characteristics but also the cellular components of blood may additionally contribute to a higher mechanical obstruction within the subarachnoid space and the glymphatic system. In addition, besides a mechanical impairment of CSF circulation, it may be suggested that blood components may exhibit toxic effects on brain tissue even at this early time point, inducing edema and further increasing ICP.

Similar to our previous studies, following blood injection CBF decreased quickly with a sustained hypoperfusion for the first hour and a recovery to a level slightly lower than baseline about 2 h later in the present study (Bach et al., 2019; Conzen et al., 2019). However, the chronological sequence of CBF differs from that of ICP and CPP, both of which deviated significantly from baseline throughout the 6-h monitoring period. Thus, hypoperfusion following SAH may not simply be an ICP- or CPP- dependent event during EBI. Interestingly, SA-CX group did only show the very early transient ischemia following the ICP peak but did not develop the prolonged hypoperfusion despite identical changes of ICP and CPP as SA-B group. In SA-CX, following the transient ischemia induced by the bolus injection, CBF rapidly recovered within less than five minutes, even generating a slight non-significant reactive hyperperfusion within this early time period. Thus, it can be speculated that blood components rather than intracranial hypertension play a more critical role in the early prolonged hypoperfusion after SAH. As far as we know, our study is the first to verify this often proposed hypothesis with our new intracranial hypertension model in rats. In the other groups of SA-C and SA-G, following the transient ischemia during the bolus injection, a rapid transient reactive hyperperfusion could be observed instead of the prolonged hypoperfusion in SA-B group, which also strengthens, at least partly, the suggested immediate and important effect of blood components in hypoperfusion after the onset of SAH. Previous results by Friedrich et al. (2012) showed the presence of constricted arterioles and microthrombosis early after the onset of SAH, which may explain the ICP-independent CBF decline. To carefully extrapolate our findings to the clinical situation, besides applying an effective ICP-management, the removal of the deposited blood may be a promising approach to attenuate or even prevent hypoperfusion already in the early phase after bleeding initiation.

Intact CA is a homeostatic process via which steady CBF is ensured over a wide range of ABP or CPP (Lassen, 1959; Fantini et al., 2016). However, in patients with aneurysmal SAH, impaired CA was found during the first 72 h and, while probably contributing to inadequate blood perfusion, is related to unfavorable prognosis (Schmieder et al., 2006; Rivera-Lara et al., 2017). Experimentally, impaired CA was also demonstrated during the first 6 h after SAH in the rat blood injection model (Jakubowski et al., 1982; Conzen et al., 2019). In the present study, at least transiently impaired CA occurred in the majority of the animals of SA-B that lasted about 2 h and subsequently returned to a level slightly below the threshold. Compared with our former study (Conzen et al., 2019), CA was only transiently and less severely impaired in the present SA-B group. Whereas in the study by Conzen et al. (2019) blood was injected by hand, in the present study a syringe pump was used with a probably more regular and less pulsatile way of infusion. Whether the way of infusion impacts CA is not clear so far and needs further evaluation. In contrast to SA-B, intact CA was maintained throughout the experiment in the majority of the animals in all other blood substitution groups. This finding indicates that the blood components may play a significant role in CA impairment after the onset of SAH which may also contribute to the early hypoperfusion. In addition, a minor contribution of the early intracranial hypertension accompanied by the transient global ischemia can also be suggested.

Recent evidence suggests that inflammatory processes, involved in many aspects of the pathological changes after SAH, play a critical role in the development of EBI (Dumont et al., 2003; Geraghty et al., 2019). Blood degradation products, such as hemoglobin, methemoglobin, heme and hemin, could be recognized by TLR 4 and subsequently initiate cytokine signaling of the inflammatory cascades (Ascenzi et al., 2005; Pradilla et al., 2010). Meanwhile, evidence also showed the involvement of elevated ICP in inflammatory events (Graetz et al., 2010). Elevated inflammatory cytokine levels in cerebrospinal fluid and systemic circulation have been proven to be correlated with the prognosis of patients with SAH (Geraghty et al., 2019). A study by Vecchione et al. (2009) also implicated TNF-α as a crucial role for the development of cerebral vasospasm after SAH, both *in vitro* and *in vivo*. Furthermore, it has been shown that experimental cerebral vasospasm can be induced by intracisternal injection of proinflammatory agents despite the absence of blood, while hypoperfusion could be ameliorated by the removal of inflammatory cells (Yanamoto et al., 1994; Nagata et al., 1996; Fujimoto et al., 2013; Neulen et al., 2019). However, controversy still exists concerning the differentiation between protective as well as destructive effects of inflammation in SAH. Besides its potential contribution to vasospasm, inflammation is also considered to be important for the clearance of free hemoglobin and for processes resulting in neural recovery (Hailer et al., 1997; Schneider et al., 2018). The bidirectional effects of inflammation after SAH seem to be depending on the timing and the surrounding environments. Our results of the cytokine increase in blood samples as well as in tissue samples of the basal cortex in the blood injection groups (SA-B, SA-B-exp2) reproduce the results of our previous study (Bach et al., 2019) and thus again confirm the published and well-known inflammatory response starting early after initiation of SAH.

Whether this initial inflammatory response is induced by blood components and very early degradation products or whether it mainly depends on the effects of the early and transient peak increase of ICP was not known so far. The present study comparing injection of different fluids lacking blood components while inducing comparable ICP courses during and after injection gives first indications toward a prominent role of the early pressure effect on the tissue. Compared with blood injection, almost the same levels of proinflammatory cytokines in blood and tissue were revealed in gelofusine and aCSF injection groups. In addition, with all substances, a substantial and almost identical ICP peak could be induced, whereas a substantial plateau level of ICP was only reached by blood and aCSF + xanthan, while injecting the low viscosity fluids a merely slightly elevated (aCSF) or physiologically normal (gelofusine) ICP level was produced. This indicates that the activated inflammatory response in the tissue may be mainly caused by the transiently elevated ICP within the first minutes inducing a short lasting transient global ischemia. In addition, from these results it can be suggested that the proinflammatory cytokines IL-6 and TNF-α and probable other inflammatory mediators within the tissue are not responsible for the tissue hypoperfusion during the first few hours after SAH because this delayed phase of hypoperfusion is lacking in animals injected with gelofusine or aCSF. The results of aCSF + xanthan injection principally support this interpretation. While perfectly modeling the ICP time course of blood injection, it induced an identical cytokine response in brain tissue but without the prolonged tissue hypoperfusion within PDP shown by blood injection.

Xanthan, a natural polysaccharide extracted mainly by bacterial fermentation from *Xanthomonas campestris*, has found wide application in food and pharmaceutical industries with respect to its unique properties, such as high viscosity, excellent thermal and pH stability, safety and biocompatibility (Kumar et al., 2018). The ability to increase the viscosity of aqueous solutions even at a very small concentration and the pseudoplastic rheological properties of xanthan revealed it suitable for us to prepare a blood analog fluid by thickening aCSF with xanthan to reach blood viscosity levels (Brookshier and Tarbell, 1993; Clauser et al., 2018). Furthermore, research on porcine coronary rings demonstrated that xanthan possesses no intrinsic vasoactive properties (van den Broek et al., 2008). Apart from the solely pressure-induced transient global ischemia caused by the strong ICP peak within the first minutes after injection start and a small transient Cushing-reflex-analog hyperperfusion around the first hour after injection, intracisternally injected aCSF + xanthan induced no significant deviation of CBF from baseline level. As injection of aCSF alone resulted in a comparable CBF course as aCSF + xanthan, it can be suggested that xanthan itself had no direct vasoactive effect on the cerebral vasculature. The analog blood flow courses in SA-C and SA-CX groups further support the major conclusion of the present study that blood components rather than ICP elevation play a critical role in the early hypoperfusion after SAH.

In contrast to the other fluids, intracisternally injected aCSF + xanthan induced a strong and very early-starting systemic inflammatory response. For xanthan a high binding affinity to Toll-like receptor 4 (TLR-4) has been described, with *in vitro* studies showing activation induced release of TNF-α by macrophages (Takeuchi et al., 2009) as well as enhanced antibody production of murine B-lymphocytes. In addition, also oral feeding studies in mice and rats suggested that dietary xanthan could trigger an inflammatory response (Ishizaka et al., 1983; Silva Rischiteli et al., 2019). It is not clear how the intracisternally injected aCSF + xanthan may have induced the systemic increase in TNF-α and IL-6, reaching blood concentrations comparable to that induced by systemic application of lipopolysaccharide (LPS) (Murray et al., 2011; Beck-Schimmer et al., 2017; Fruekilde et al., 2022). A transport of xanthan from the subarachnoid space along the perivascular glymphatic pathways with final drainage out of the brain into the nasal and cervical lymphatic system seems reasonable [for overview (see Iliff et al., 2012; Jessen et al., 2015; Wardlaw et al., 2020)], finally activating macrophages and other immune cells within the lymph nodes. It may be suggested that the proposed restriction of xanthan to the perivascular space on its way outside of the brain without direct contact to the local immune cells within the parenchyma may have prevented activation of microglial cells and thus a local inflammatory response by xanthan at this early time point. As mentioned above, the systemically enhanced proinflammatory cytokines are unlikely to be involved in the early prolonged hypoperfusion. On the other hand, the strong systemic inflammation may have induced vasodilation which then may have counteracted vasoconstrictive mechanisms in SA-CX animals, possibly masking an ICP-induced prolonged hypoperfusion also in SA-CX. Whether systemic inflammation has acute vasodilative effects is still controversly discussed, with some studies describing no or only anecdotal findings of vasodilation (Brezzo et al., 2020; Manouchehrian et al., 2021; Fruekilde et al., 2022) while others reporting an increase in resting CBF (however, accompanied by a decrease in ABP, which did not occur in our study) by systemically applied LPS (Rosengarten et al., 2007). We only detected a small and transient increase of CBF within the first hour after CX injection which may be related to the systemic inflammatory response. At the 6 h time point after blood injection, hypoperfusion has been dissipated and CBF was returned to baseline level, but we did not find a permanently elevated CBF after aCSF + xanthan injection which would have been expected by the still significantly elevated cytokine levels in case of a proposed effect on resting CBF. Based on the controversial data in the literature and our findings of identical CBF level of SA-CX and SA-B at the later time points up to 6 h compared with the respective baseline despite higher systemic cytokine concentrations in SA-CX, it seems unlikely that the systemic inflammation following intracisternal aCSF + xanthan injection induced an increase in resting CBF in our study.

Our experiment with aCSF + xanthan injection further indicates that systemic inflammation alone may not be responsible for a prolonged hypoperfusion during the first few hours after SAH. Moreover, a study by Liu et al. (2017) revealed a two-way immunomodulatory effect of xanthan on macrophages since an inflammatory response was triggered by the sole effect of xanthan but was significantly suppressed while combined with LPS. In addition, other experimental studies have demonstrated protective effects of xanthan on controlling osteoarthritis and temporomandibular disorder by suppressing inflammation (Han et al., 2012; Li et al., 2019; Yuan et al., 2020). It may thus be interesting to conduct further research on the inflammatory response when xanthan is applied in SAH models. Moreover, antioxidant effect is also conferred to xanthan due to its abundant in OH- residues. The amelioration of dry eye syndrome after the treatment with xanthan seemed to benefit from its antioxidant activity (Amico et al., 2015; Postorino et al., 2020). Considering the crucial roles of inflammation and oxidation in the pathological process after SAH, it will be also interesting to study the possible protective role of xanthan in experimental SAH models.

## 5. Limitations of the present study and suggestions for further research

First, xanthan is not as inert as expected due to the strongly activated systemic inflammation. However, our results indicated that it did not affect CBF significantly, and thus making or conclusion still valid that blood components rather than elevated ICP may play a critical role for early hypoperfusion events in EBI processes. Moreover, as mentioned above, our results revealed some other interesting points about inflammation and xanthan, uncovering a possibly minor role of systemic inflammatory responses for vasospasm related events. A further refinement with substitution of xanthan by another, more biocompatible substance for imitation of ICP according to the course after blood injection may confirm the results of the present study. Second, our aCSF + xanthan fluid showed comparable viscosity as blood, however, it still lacked blood cell analog particles which may result in different shear properties and local rheologic performance within the irregularly and complex morphology of the subarachnoid space. Therefore, it will be interesting to add inert microparticles with the same size, deformation properties and amount as blood cells to better mimic blood distribution within the subarachnoid space. Third, the still needed slightly higher injection volume of aCSF + xanthan compared with blood-injection may have led to a partial outwash of pathophysiologically relevant vasoactive mediators. Adding microparticles in blood-viscosity comparable fluids may also further decrease the need for higher infusion volume for ICP simulation. Fourth, a comparable distribution of aCSF + xanthan compared with blood within the subarachnoid space was not tested in the present study. Theoretically, the distribution of this blood-substitution fluid may follow mainly the way of the non-corpuscular fraction of blood, however, lacking the effects of a possible blockade probably built up by red blood cells within the subarachnoid space. To exactly illustrate and compare the distribution of aCSF + xanthan with that of blood, in a further study using dye-labeled xanthan (or any other ideal substitute) added to aCSF will provide a tool to investigate its exact distribution in comparison with blood injection using labeled red blood cells and plasma. Finally, the injection volume or rate of infusion of aCSF + xanthan, instead of being set at a fixed value, was slightly adjusted throughout each experiment depending on the individual ICP course, which may also increase difficulty on the repetition of experiments especially for personnel with less experience.

## 6. Conclusion

To differentiate the role of ICP elevation compared with blood components and their degradation products for early pathophysiology in SAH, on the basis of the well-known cisterna magna blood injection model for SAH in rats, we established a subarachnoid-volume occupying model by cisterna magna injection of blood substitution fluids that exactly mimicked the course of ICP in the first 6 h of the original model. While simultaneously analyzing the hemodynamic course within the microcirculation, we demonstrate that fluids other than blood do not induce the typical prolonged hypoperfusion elicited following blood-injection despite comparable ICP progress. Our study strongly suggests that blood components rather than elevated ICP play an important role for early hypoperfusion events in PDP. Moreover, our results also, at least partly, demonstrate that the well-known SAH-associated systemic inflammation alone may not affect cerebral vascular tone, and especially the early peak of intracranial hypertension after SAH initiation may be the main trigger for the inflammatory response during the first few hours after SAH. In contrast to the systemic inflammation, the inflammatory response in brain tissue following the injection of all blood substitution fluids was comparable to blood injection and to each other regardless of the type of the fluid, making the early tissue inflammation unlikely to be involved as an important mechanism of the early prolonged hypoperfusion within the first 6 h after experimental SAH. Xanthan supplementation to aCSF for viscosity enhancement caused a significantly stronger systemic inflammatory reaction than all other fluids including blood, recommending the search for another, more inert and biologically inactive thickener for water-based substitution fluids in further studies.

## Data availability statement

The raw data supporting the conclusions of this article will be made available by the authors, without undue reservation.

## Ethics statement

The animal study was reviewed and approved by the Landesamt für Natur, Umwelt und Verbraucherschutz (LANUV) Nordrhein–Westfalen, Recklinghausen, Germany; AZ 84-02.04.2015.A412.

## Author contributions

GH, CC-D, and UL conceived and designed the experiments and the study protocol. GH and CC-D performed the experiments. GH and UL analyzed the data and wrote the manuscript. GH, CC-D, TS, GS, and UL interpretation of the data. EH designed and performed enzyme-linked immunosorbent assays. MS, JC, and GH designed and performed viscosity analysis. All authors critically reviewed the manuscript and approved the submitted version.
