# Supplementary material for: Effect of isolated intracranial hypertension on cerebral perfusion within the phase of primary disturbances after subarachnoid hemorrhage in rats
Source: Front Cell Neurosci. 2023 Jul 12;17:1115385. doi: 10.3389/fncel.2023.1115385 (PMC10368889; doi:10.3389/fncel.2023.1115385)
Supplement: Supplementary file 1 [file Data_Sheet_1.pdf]

Supplementary Material

Supplementary Methods

Experimental design

Experimental study 1:

70 rats were allocated to 5 different groups with a monitoring period of 6 hours

1) SA-B group (n=16): 0.5 ml heparinized autologous arterial blood (80 IU heparin) was injected into cisterna magna within 1 minute.

2) SA-G group (n=16): 0.5 ml gelofusine (4%) was injected into cisterna magna within 1 minute.

3) SA-S group (n=6): 0.5 or 0.3 ml silicone oil was injected into cisterna magna within 1 minute. Three types of silicone oil with different viscosity levels (5, 3 and 1) were tried successively. This study was discontinued due to spontaneous death of all except one animal shortly after injection.

4) SA-C group (n=16): 1.5 ml aCSF was injected into cisterna magna within the first minute followed by continuous infusion at the rate of 2 ml/h till the end of the experiment. Based on published data of total intracranial CSF volume of about 200-300 µl and a mean flow rate of approximately 3 µl/min in young rats[1, 2], the maximum infusion rate was set at 2 ml/h, representing appropriately 10 times the physiological CSF exchange rate. Recipe of aCSF: aCSF was mixed by solution A (8.66g NaCl, 0.206g $CaCl_2 \cdot 2H_2O$, 0.224g KCl and 0.163g $MgCl_2 \cdot 6H_2O$ dissolved in 500 ml pyrogen-free, sterile water) and B (0.214g $Na_2HPO_4 \cdot 7H_2O$ and 0.027g $NaH_2PO_4 \cdot H_2O$ dissolved in 500 ml pyrogen-free, sterile water) in a ratio of 1:1 (all chemicals were purchased from Sigma-Aldrich).

5) SA-CX group (n=16): CX is a solution of aCSF plus xanthan, with final xanthan concentration of 0.1%. 1.0-1.2 ml CX was injected into cisterna magna within the first minute followed by continuous infusion during the first 3 hours with the rate adjusted according to the individual ICP course (rate range: 0 - 1 ml/h during first 30 minutes followed by 0 - 0.1 ml/h thereafter).

Experimental study 2:

12 rats were allocated to 2 different groups with a monitoring period of 2 hours

1) SA-B-exp2 group (n=4): 0.5 ml heparinized autologous arterial blood (80 IU heparin) was injected into cisterna magna within 1 minute.
2) SA-CX-exp2 group (n=8): 1.0-1.3 ml CX was injected into cisterna magna within the first minute followed by continuous infusion with the rate adjusted according to the individual ICP course (rate range: 0 - 1 ml/h during first 30 minutes followed by 0 - 0.1 ml/h thereafter.

Surgery - detailed description:

Following successful anesthesia induction by inhalation of 4% isoflurane mixed with O_2 and N_2O, appropriate anesthesia for the surgery was achieved with isoflurane set at 1.6-2% according to the response to toe pinch frequently checked. Intravenous fentanyl infusion (0.015mg/kg/h) via a femoral vein catheter together with additional subcutaneously injected ropivacaine at each skin incision were performed for analgesia. Body temperature, measured through a rectal probe, was maintained at around 37℃ by employing a servo-controlled heating pad (Harvard Apparatus Ltd., Kent, England). Tracheotomy was performed for artificial ventilation with ventilator parameters adjusted according to data from blood gas analysis (BGA) to keep systemic arterial pO_2, pCO_2 and pH in the physiological range. Catheterizations of femoral vein and artery were then performed for administration of fentanyl, for continuous arterial blood pressure (ABP) monitoring (BLPR2 and SYS-BP1, WPI, Friedberg, Germany) and for hourly BGA measurement (Eschweiler combi line, Eschweiler GmBH & Co KG, Kiel, Germany), respectively.

Next, the rat was placed prone on a stereotactic apparatus (Stereotaxic Frame, WPI, Friedberg, Germany). For measurement of local cerebral blood flow (CBF), a cranial bone window (5 mm × 8 mm, medial and rear borders were 2.5 mm lateral to midline and 4 mm posterior to bregma) over the right parietal cortex was drilled out under continuous saline irrigation till the pial vessels were clearly visible, while the thinned bone layer was preserved intact. The bone window was then encircled by bone wax, filled with saline and enclosed by an appropriate cover glass. Electroencephalogram (EEG), not analyzed in the present study, was monitored with a silver wire electrode placed at the epidural space 2 mm posterior to the cranial window and a reference electrode placed subcutaneously on the back of the neck. A microcatheter, advanced into the cisterna magna, was used to inject blood or different blood substitutes as

planned. A needle, connected to the pressure monitor (BLPR2 and SYS-BP1, WPI, Friedberg, Germany) through a saline-filled microcatheter for the measurement of ICP, was inserted into the subarachnoid space through the atlantooccipital membrane.

References

1. Chiu C, Miller MC, Caralopoulos IN, Worden MS, Brinker T, Gordon ZN, et al. Temporal course of cerebrospinal fluid dynamics and amyloid accumulation in the aging rat brain from three to thirty months. Fluids Barriers CNS. 2012;9:3
2. Murtha LA, Yang Q, Parsons MW, Levi CR, Beard DJ, Spratt NJ, et al. Cerebrospinal fluid is drained primarily via the spinal canal and olfactory route in young and aged spontaneously hypertensive rats. Fluids Barriers CNS. 2014;11:12

Supplementary figures:

Supplementary Figure S1: Images of representative brains from experimental study 1: In SA-B group, blood was distributed at the basal cistern and the surface of the hemisphere, while there was no subarachnoid blood in all other groups.

Supplementary Figure S2: Example of initial ICP time courses of silicone oil compared with blood injection. Even with high-viscosity silicone oil, peak ICP remained lower than that of blood injection.

Supplementary Figure S3: ICP - within group analysis: Quantitative analysis at distinct time points (**** $p<0.0001$; *** $p < 0.001$; ** $p < 0.01$; * $p < 0.05$)

Supplementary Figure S4: Arterial blood pressure (ABP). (A) ABP courses induced by the fluids throughout the measurement of 6 hours, insert: first 10 minutes enlarged; **(B)** quantitative analysis at distinct time points – between group analysis; **(C)** quantitative analysis at distinct time points – within group analysis. (**** $p<0.0001$; *** $p < 0.001$; ** $p < 0.01$; * $p < 0.05$)

Supplementary Figure S5: Cerebral perfusion pressure (CPP). (A) CPP courses induced by the fluids throughout the measurement of 6 hours, insert: first 10 minutes enlarged; **(B)** quantitative analysis at distinct time points – between group analysis; **(C)** quantitative analysis at distinct time points – within group analysis. (**** $p<0.0001$; *** $p < 0.001$; ** $p < 0.01$; * $p < 0.05$)

Supplementary Figure S6: CBF - within group analysis. quantitative analysis at distinct time points (**** $p<0.0001$; *** $p < 0.001$; ** $p < 0.01$; * $p < 0.05$)

Supplementary Figure S7: PRx as measure for cerebral autoregulation following fluid injection. Single courses of animals within each group: **(A)** all within one figure or distributed between categories of **(B)** intact or **(C)** transiently or **(D)** mostly impaired CA according to the cutoff value of PRx 0.2. **(E)** depicts the median course of all animal within each group supplemented with the data range presented as the shaded area.
