# Supplementary material for: Effect of isolated intracranial hypertension on cerebral perfusion within the phase of primary disturbances after subarachnoid hemorrhage in rats
Source: Front Cell Neurosci. 2023 Jul 12;17:1115385. doi: 10.3389/fncel.2023.1115385 (PMC10368889; doi:10.3389/fncel.2023.1115385)
Supplement: Supplementary file 2 [file Data_Sheet_2.PDF]

Table S1: ICP at different time points

data expressed as median with 3. quartile (Q3) and 1.quartile (Q1); differences between groups and deviation from baseline within groups were analyzed by two-way ANOVA followed by Dunnett's multiple comparisons test; bl=baseline

ICP in mmHg

	SA-B				SA-C				SA-CX				SA-G			
	median	Q3	Q1	p vs bl	median	Q3	Q1	p vs bl	median	Q3	Q1	p vs bl	median	Q3	Q1	p vs bl
**baseline**	11.8	13.8	10.9		12.1	12.8	11.5		12.2	12.7	11.7		10.5	11.6	8.3	
**peak**	124.6	159.2	108.0	<0.0001	135.7	162.9	102.2	<0.0001	137.6	189.3	101.1	<0.0001	89.4	111.7	61.0	<0.0001
**2min**	81.8	102.9	63.8	<0.0001	30.3	34.5	26.7	<0.0001	96.1	106.1	59.3	<0.0001	25.3	32.2	18.9	<0.0001
**5min**	32.5	44.0	24.0	0.0005	19.5	20.2	17.8	<0.0001	42.7	55.9	22.9	0.0002	14.1	15.7	12.5	0.0035
**30min**	23.8	35.0	17.1	0.0031	20.1	22.3	17.7	<0.0001	34.2	34.7	28.9	<0.0001	11.9	14.9	10.7	0.1953
**60min**	30.4	42.4	20.4	0.0006	20.1	21.6	17.0	<0.0001	32.9	35.5	31.2	<0.0001	11.9	13.7	10.2	0.4197
**120min**	29.3	43.0	21.4	0.0005	20.5	22.4	18.0	0.0003	36.0	38.1	28.7	<0.0001	12.1	13.2	9.9	0.5784
**240min**	22.8	35.5	18.9	0.0009	26.9	28.3	20.0	<0.0001	29.2	32.8	25.6	<0.0001	10.7	13.1	9.8	0.9187
**360min**	20.8	28.9	16.8	0.0008	25.6	29.4	20.0	0.0002	28.4	31.6	26.2	<0.0001	10.4	13.2	9.4	0.9746

**baseline**	**p**
SA-B vs. SA-C	0.8967
SA-B vs. SA-CX	0.9093
SA-B vs. SA-G	0.1882
**peak**	
SA-B vs. SA-C	0.9901
SA-B vs. SA-CX	0.9827
SA-B vs. SA-G	0.0017
**2min**	
SA-B vs. SA-C	<0.0001
SA-B vs. SA-CX	0.9977
SA-B vs. SA-G	<0.0001

**5min**	**p**
SA-B vs. SA-C	0.0052
SA-B vs. SA-CX	0.7675
SA-B vs. SA-G	0.0007
**30min**	
SA-B vs. SA-C	0.1787
SA-B vs. SA-CX	0.6585
SA-B vs. SA-G	0.0016
**60min**	
SA-B vs. SA-C	0.0119
SA-B vs. SA-CX	>0.9999
SA-B vs. SA-G	0.0002

**120min**	**p**
SA-B vs. SA-C	0.0179
SA-B vs. SA-CX	>0.9999
SA-B vs. SA-G	0.0001
**240min**	
SA-B vs. SA-C	0.9961
SA-B vs. SA-CX	0.8643
SA-B vs. SA-G	<0.0001
**360min**	
SA-B vs. SA-C	0.8242
SA-B vs. SA-CX	0.3016
SA-B vs. SA-G	<0.0001

Table S2: CBF at different time points

data expressed as median with 3. quartile (Q3) and 1.quartile (Q1); differences between groups and deviation from baseline within groups were analyzed by two-way ANOVA followed by Dunnett's multiple comparisons test; bl=baseline

CBF in % (with 5min-average before fluid injection set as 100%)

	SA-B				SA-C				SA-CX				SA-G			
	**median**	**Q3**	**Q1**	**p (vs bl)**	**median**	**Q3**	**Q1**	**p (vs bl)**	**median**	**Q3**	**Q1**	**p (vs bl)**	**median**	**Q3**	**Q1**	**p (vs bl)**
**baseline**	99.6	101.8	98.7		100.4	102.6	98.5		100.3	102.0	99.3		98.8	102.8	96.8	
**minimum**	9.2	13.6	7.2	<0.0001	7.4	26.8	4.8	<0.0001	7.3	20.3	5.0	<0.0001	26.0	53.9	20.4	<0.0001
**2min**	42.6	64.3	20.6	0.026	208.6	242.4	151.3	0.0004	101.6	125.4	70.7	0.9977	170.5	201.0	117.7	0.0005
**5min**	73.6	91.5	41.2	0.0362	125.2	143.0	109.2	0.0045	105.8	133.0	98.7	0.1364	118.4	125.9	108.0	0.0019
**30min**	59.1	94.1	32.0	0.0011	93.8	100.4	86.3	0.921	99.9	111.9	89.3	>0.9999	115.4	122.9	102.2	0.0554
**60min**	62.3	102.4	35.5	0.0424	105.9	108.9	93.1	0.9756	132.0	149.2	117.8	0.0002	106.9	117.6	99.6	0.6286
**120min**	83.4	107.6	51.1	0.38	98.0	119.5	83.5	0.9603	114.7	141.5	100.0	0.1795	118.8	123.9	103.2	0.1746
**240min**	81.0	112.0	51.5	0.4775	121.3	148.3	88.0	0.8537	117.3	127.0	98.9	0.2771	113.8	130.1	98.4	0.3415
**360min**	74.3	97.5	57.5	0.6901	102.0	136.2	82.4	0.9997	116.6	128.0	102.9	0.2	122.9	133.6	104.7	0.2237

**baseline**	**p**
SA-B vs. SA-C	0.8631
SA-B vs. SA-CX	0.6906
SA-B vs. SA-G	0.7847
**minimum**	
SA-B vs. SA-C	0.5754
SA-B vs. SA-CX	0.7584
SA-B vs. SA-G	0.0057
**2min**	
SA-B vs. SA-C	<0.0001
SA-B vs. SA-CX	0.0183
SA-B vs. SA-G	<0.0001

**5min**	**p**
SA-B vs. SA-C	0.0001
SA-B vs. SA-CX	0.0017
SA-B vs. SA-G	0.0006
**30min**	
SA-B vs. SA-C	0.0026
SA-B vs. SA-CX	0.0007
SA-B vs. SA-G	<0.0001
**60min**	
SA-B vs. SA-C	0.0167
SA-B vs. SA-CX	<0.0001
SA-B vs. SA-G	0.0095

**120min**	**p**
SA-B vs. SA-C	0.2036
SA-B vs. SA-CX	0.029
SA-B vs. SA-G	0.0404
**240min**	
SA-B vs. SA-C	0.1934
SA-B vs. SA-CX	0.0508
SA-B vs. SA-G	0.0641
**360min**	
SA-B vs. SA-C	0.7794
SA-B vs. SA-CX	0.1067
SA-B vs. SA-G	0.0715

Table S3: ABP at different time points

data expressed as median with 3. quartile (Q3) and 1.quartile (Q1); differences between groups and deviation from baseline within groups were analyzed by Mixed-effects model (REML) followed by Dunnett's multiple comparisons test; bl=baseline

ABP in mmHg

	SA-B				SA-C				SA-CX				SA-G			
	median	Q3	Q1	p (vs bl)	median	Q3	Q1	p (vs bl)	median	Q3	Q1	p (vs bl)	median	Q3	Q1	p (vs bl)
**baseline**	70.2	74.4	66.5		67.4	71.7	64.2		68.8	72.9	66.8		70.2	71.6	65.4	
**peak**	136.7	150.8	119.4	<0.0001	133.1	147.3	115.2	<0.0001	143.5	157.9	113.9	<0.0001	105.4	121.6	90.8	<0.0001
**2min**	114.1	136.5	105.2	<0.0001	97.2	111.2	91.8	<0.0001	117.2	126.2	97.6	<0.0001	92.3	104.7	82.4	<0.0001
**5min**	91.9	98.5	78.1	0.0034	68.4	85.6	64.4	0.5013	81.3	91.2	69.5	0.0425	83.3	93.5	78.8	0.0015
**30min**	65.2	71.2	61.9	0.1306	63.0	68.7	60.6	0.252	70.9	77.0	68.7	0.1696	73.0	77.7	67.4	0.1229
**60min**	68.3	75.8	62.4	0.9994	65.2	69.2	62.9	0.9533	80.6	84.1	75.3	0.0004	66.9	70.6	65.4	0.9994
**120min**	73.4	79.7	66.7	0.932	69.4	72.3	64.4	0.9068	75.1	79.1	70.5	0.0757	66.9	69.1	65.6	0.9996
**240min**	66.9	71.9	62.1	0.9057	73.0	78.4	69.9	0.1268	66.7	69.7	61.7	0.1735	65.6	67.7	63.2	0.8758
**360min**	66.8	69.0	62.0	0.7532	72.5	77.9	65.9	0.2352	67.1	73.1	61.4	0.2641	67.1	72.7	64.7	0.9862

**baseline**	**p**
SA-B vs. SA-C	0.95
SA-B vs. SA-CX	0.913
SA-B vs. SA-G	0.9997
**peak**	
SA-B vs. SA-C	0.9858
SA-B vs. SA-CX	0.9983
SA-B vs. SA-G	0.0006
**2min**	
SA-B vs. SA-C	0.0252
SA-B vs. SA-CX	0.6346
SA-B vs. SA-G	0.002

**5min**	**p**
SA-B vs. SA-C	0.144
SA-B vs. SA-CX	0.3895
SA-B vs. SA-G	0.8253
**30min**	
SA-B vs. SA-C	0.9828
SA-B vs. SA-CX	0.0212
SA-B vs. SA-G	0.0224
**60min**	
SA-B vs. SA-C	0.6605
SA-B vs. SA-CX	0.0791
SA-B vs. SA-G	0.9996

**120min**	**p**
SA-B vs. SA-C	0.9966
SA-B vs. SA-CX	0.7472
SA-B vs. SA-G	0.7988
**240min**	
SA-B vs. SA-C	0.143
SA-B vs. SA-CX	0.9996
SA-B vs. SA-G	0.9827
**360min**	
SA-B vs. SA-C	0.0672
SA-B vs. SA-CX	0.9634
SA-B vs. SA-G	0.5935

Table S4: CPP at different time points

data expressed as median with 3. quartile (Q3) and 1.quartile (Q1); differences between groups and deviation from baseline within groups were analyzed by Mixed-effects model (REML) followed by Dunnett's multiple comparisons test; bl=baseline

CPP in mmHg

	SA-B				SA-C				SA-CX				SA-G			
	median	Q3	Q1	p (vs bl)	median	Q3	Q1	p (vs bl)	median	Q3	Q1	p (vs bl)	median	Q3	Q1	p (vs bl)
**baseline**	58.4	62.1	51.7		55.6	59.6	52.7		57.7	60.5	54.8		59.1	63.4	54.5	
**minimum**	-6.9	5.8	-20.5	<0.0001	-9.5	6.7	-32.6	<0.0001	-3.3	9.2	-37.3	<0.0001	9.0	18.0	3.7	<0.0001
**2min**	34.4	47.4	26.9	0.0003	70.3	75.4	61.1	0.0004	24.8	43.3	15.6	<0.0001	72.2	76.2	62.9	0.0395
**5min**	52.3	73.3	39.9	0.9947	48.7	66.4	44.8	0.9998	39.7	48.5	34.6	<0.0001	68.1	78.9	65.9	0.025
**30min**	33.1	52.7	29.3	0.0002	44.0	46.7	40.1	<0.0001	39.0	47.9	35.6	<0.0001	60.0	64.2	55.5	0.8688
**60min**	32.8	53.4	24.7	0.0036	46.5	49.3	42.7	0.0037	50.0	51.7	43.2	<0.0001	56.1	59.0	52.6	0.9979
**120min**	35.5	49.8	29.2	0.0009	48.5	53.3	44.3	0.1837	41.4	46.9	35.3	<0.0001	55.4	57.1	52.9	0.9195
**240min**	37.2	49.3	32.3	0.0004	46.0	51.6	43.9	0.0212	38.0	42.4	31.3	<0.0001	55.4	56.4	51.8	0.6483
**360min**	40.8	47.0	36.7	0.001	48.8	56.9	37.5	0.1038	37.8	41.0	35.3	<0.0001	58.8	62.6	51.7	0.9995

**baseline**	**p**
SA-B vs. SA-C	0.9944
SA-B vs. SA-CX	0.748
SA-B vs. SA-G	0.5716
**minimum**	
SA-B vs. SA-C	0.9222
SA-B vs. SA-CX	0.8931
SA-B vs. SA-G	0.0219
**2min**	
SA-B vs. SA-C	<0.0001
SA-B vs. SA-CX	0.2807
SA-B vs. SA-G	<0.0001

**5min**	**p**
SA-B vs. SA-C	0.9624
SA-B vs. SA-CX	0.1019
SA-B vs. SA-G	0.0326
**30min**	
SA-B vs. SA-C	0.2443
SA-B vs. SA-CX	0.5552
SA-B vs. SA-G	<0.0001
**60min**	
SA-B vs. SA-C	0.1854
SA-B vs. SA-CX	0.0982
SA-B vs. SA-G	0.0018

**120min**	**p**
SA-B vs. SA-C	0.0465
SA-B vs. SA-CX	0.7869
SA-B vs. SA-G	0.0005
**240min**	
SA-B vs. SA-C	0.152
SA-B vs. SA-CX	0.8767
SA-B vs. SA-G	0.0002
**360min**	
SA-B vs. SA-C	0.6137
SA-B vs. SA-CX	0.7061
SA-B vs. SA-G	<0.0001

Table S5: PRx at different time points

data expressed as median with 3. quartile (Q3) and 1.quartile (Q1); differences between groups and deviation from baseline within groups were analyzed by two-way ANOVA followed by Dunnett's multiple comparisons test; bl=baseline

PRx

	SA-B				SA-C				SA-CX				SA-G			
	median	Q3	Q1	p (vs each)	median	Q3	Q1	p (vs each)	median	Q3	Q1	p (vs each)	median	Q3	Q1	p (vs each)
**30min**	0.09	0.31	0.01	>0.05	-0.03	0.07	-0.22	>0.05	-0.01	0.07	-0.08	>0.05	0.02	0.06	0.01	>0.05
**60min**	0.19	0.33	0.03		-0.08	0.10	-0.20		-0.01	0.11	-0.12		0.04	0.07	0.02	
**120min**	0.10	0.37	0.04		-0.08	0.10	-0.13		-0.05	0.06	-0.13		0.01	0.05	-0.02	
**150min**	0.23	0.37	0.00		-0.01	0.17	-0.09		-0.06	0.08	-0.14		0.01	0.03	0.00	
**240min**	0.10	0.25	-0.02		-0.05	0.17	-0.12		-0.04	0.08	-0.13		0.00	0.05	-0.02	
**330min**	0.04	0.21	-0.01		0.00	0.09	-0.13		-0.09	-0.01	-0.19		0.00	0.06	-0.02	

**30min**	**p**	**120min**	**p**	**240min**	
SA-B vs. SA-C	0.0536	SA-B vs. SA-C	0.0327	SA-B vs. SA-C	0.1959
SA-B vs. SA-CX	0.0759	SA-B vs. SA-CX	0.027	SA-B vs. SA-CX	0.0365
SA-B vs. SA-G	0.2123	SA-B vs. SA-G	0.058	SA-B vs. SA-G	0.1331
**60min**		**150min**	**p**	**330min**	**p**
SA-B vs. SA-C	0.028	SA-B vs. SA-C	0.0673	SA-B vs. SA-C	0.3638
SA-B vs. SA-CX	0.0344	SA-B vs. SA-CX	0.0061	SA-B vs. SA-CX	0.0114
SA-B vs. SA-G	0.0807	SA-B vs. SA-G	0.018	SA-B vs. SA-G	0.2696

Table S6: IL-6 from blood samples at different time points

data expressed as median with 3. quartile (Q3) and 1.quartile (Q1); for SA-B–exp2 and SA-CX-exp2-Data, differences between groups were analyzed by two-way ANOVA followed by Šídák's multiple comparisons test and deviation from baseline within groups were analyzed by two-way ANOVA followed Dunnett's multiple comparisons test; for SA-B, SA-C and SA-CX data differences between groups were analyzed by ordinary one-way ANOVA followed by Dunnett's multiple comparisons test; bl=baseline

IL-6 in pg/ml

	SA-B-exp2				SA-CX-exp2			
	median	Q3	Q1	p (vs bl)	median	Q3	Q1	p (vs bl)
**baseline**	33	70	23		76	138	46	
**30min**	155	162	62	0.1594	325	455	160	0.0178
**60min**	195	255	132	0.0466	5 386	7 200	4 001	0.0009
**90min**	232	282	144	0.0535	30 997	37 516	23 577	0.001
**120min**	289	319	220	0.0136	66 425	83 316	39 129	0.0031

	SA-B			SA-CX			SA-C		
	median	Q3	Q1	median	Q3	Q1	median	Q3	Q1
**360min**	535	630	422	15 671	60 116	4 244	800	1 007	290

**baseline**	**p**
SA-B-exp2 vs. SA-CX-exp2	0.5335
**30min**	**p**
SA-B-exp2 vs. SA-CX-exp2	0.188

**60min**	**p**
SA-B-exp2 vs. SA-CX-exp2	0.0017
**90min**	**p**
SA-B-exp2 vs. SA-CX-exp2	0.0017

**120min**	**p**
SA-B-exp2 vs. SA-CX-exp2	0.0052
**360min**	**p**
SA-B vs. SA-C	0.9999
SA-B vs. SA-CX	0.0398

Table S7: TNF-α from blood samples at different time points

data expressed as median with 3. quartile (Q3) and 1.quartile (Q1); for SA-B–exp2 and SA-CX-exp2-Data, differences between groups were analyzed by two-way ANOVA followed by Šídák's multiple comparisons test and deviation from baseline within groups were analyzed by two-way ANOVA followed Dunnett's multiple comparisons test; for SA-B, SA-C and SA-CX data differences between groups were analyzed by ordinary one-way ANOVA followed by Dunnett's multiple comparisons test; bl=baseline

TNF-α in pg/ml

	SA-B-exp2				SA-CX-exp2							
	median	Q3	Q1	p (vs bl)	median	Q3	Q1	p (vs bl)				
**baseline**	50	99	1		30	41	18					
**30min**	151	377	-76	0.4099	1 288	1 922	654	0.0141				
**60min**	123	270	-25	0.28	23 266	36 976	9 556	0.0253				
**90min**	95	203	-13	0.324	41 298	48 997	33 599	0.0003				
**120min**	85	173	-3	0.3295	28 496	48 015	8 977	0.0418				
	**SA-B**				**SA-CX**				**SA-C**			
	**median**	**Q3**	**Q1**		**median**	**Q3**	**Q1**		**median**	**Q3**	**Q1**	
**360min**	24	41	8		609	1 205	13		25	34	15	

**baseline**	**p**	**60min**	**p**	**120min**	**p**
SA-B-exp2 vs. SA-CX-exp2	0.8131	SA-B-exp2 vs. SA-CX-exp2	0.0461	SA-B-exp2 vs. SA-CX-exp2	0.0755
**30min**	**p**	**90min**	**p**	**360min**	**p**
SA-B-exp2 vs. SA-CX-exp2	0.0286	SA-B-exp2 vs. SA-CX-exp2	0.0006	SA-B vs. SA-C	>0.9999
				SA-B vs. SA-CX	0.0306

Table S8: IL-6 from tissue samples

data expressed as median with 3. quartile (Q3) and 1.quartile (Q1); differences between selected groups were analyzed by ordinary one-way ANOVA followed by Šídák's multiple comparisons test; bl=baseline

IL-6 in pg/g protein

	parietal cortex			basal cortex		
	median	Q3	Q1	median	Q3	Q1
**SA-B-exp2 (2h)**	182	187	178	130	144	113
**SA-CX-exp2 (2h)**	165	183	148	208	306	92
**SA-B (6h)**	194	201	175	305	323	269
**SA-C (6h)**	191	212	178	310	340	276
**SA-CX (6h)**	200	207	190	277	328	268
**SA-G (6h)**	n.d.	n.d.	n.d.	254	266	224

**parietal cortex**	**p**
SA-B-exp2 vs. SA-CX-exp2	0.8343
SA-B-exp2 vs. SA-B	0.9923
SA-CX-exp2 vs. SA-CX	0.1122
SA-B vs. SA-C	0.9979
SA-B vs. SA-CX	0.9584

**basal cortex**	**p**
SA-B-exp2 vs. SA-CX-exp2	0.4204
SA-B-exp2 vs. SA-B	0.002
SA-CX-exp2 vs. SA-CX	0.7317
SA-B vs. SA-C	>0.9999
SA-B vs. SA-CX	0.7256
SA-B vs. SA-G	0.9999

Table S9: TNF-α from tissue samples

data expressed as median with 3. quartile (Q3) and 1.quartile (Q1); differences between selected groups were analyzed by ordinary one-way ANOVA followed by Šídák's multiple comparisons test; bl=baseline

TNF-α in pg/g protein

	parietal cortex			basal cortex		
	median	Q3	Q1	median	Q3	Q1
**SA-B-exp2 (2h)**	561	594	549	398	415	379
**SA-CX-exp2 (2h)**	578	675	561	448	739	421
**SA-B (6h)**	573	578	566	716	790	647
**SA-C (6h)**	576	582	561	789	795	753
**SA-CX (6h)**	634	645	601	712	773	665
**SA-G (6h)**	n.d.	n.d.	n.d.	817	842	742

parietal cortex	p
SA-B-exp2 vs. SA-CX-exp2	0.3154
SA-B-exp2 vs. SA-B	>0.9999
SA-CX-exp2 vs. SA-CX	0.9431
SA-B vs. SA-C	>0.9999
SA-B vs. SA-CX	0.1166

basal cortex	p
SA-B-exp2 vs. SA-CX-exp2	0.0944
SA-B-exp2 vs. SA-B	0.0002
SA-CX-exp2 vs. SA-CX	0.0007
SA-B vs. SA-C	>0.9999
SA-B vs. SA-CX	0.7221
SA-B vs. SA-G	0.9051
