# Supplementary figures and images for: Effect of isolated intracranial hypertension on cerebral perfusion within the phase of primary disturbances after subarachnoid hemorrhage in rats
Source: Front Cell Neurosci. 2023 Jul 12;17:1115385. doi: 10.3389/fncel.2023.1115385 (PMC10368889; doi:10.3389/fncel.2023.1115385)

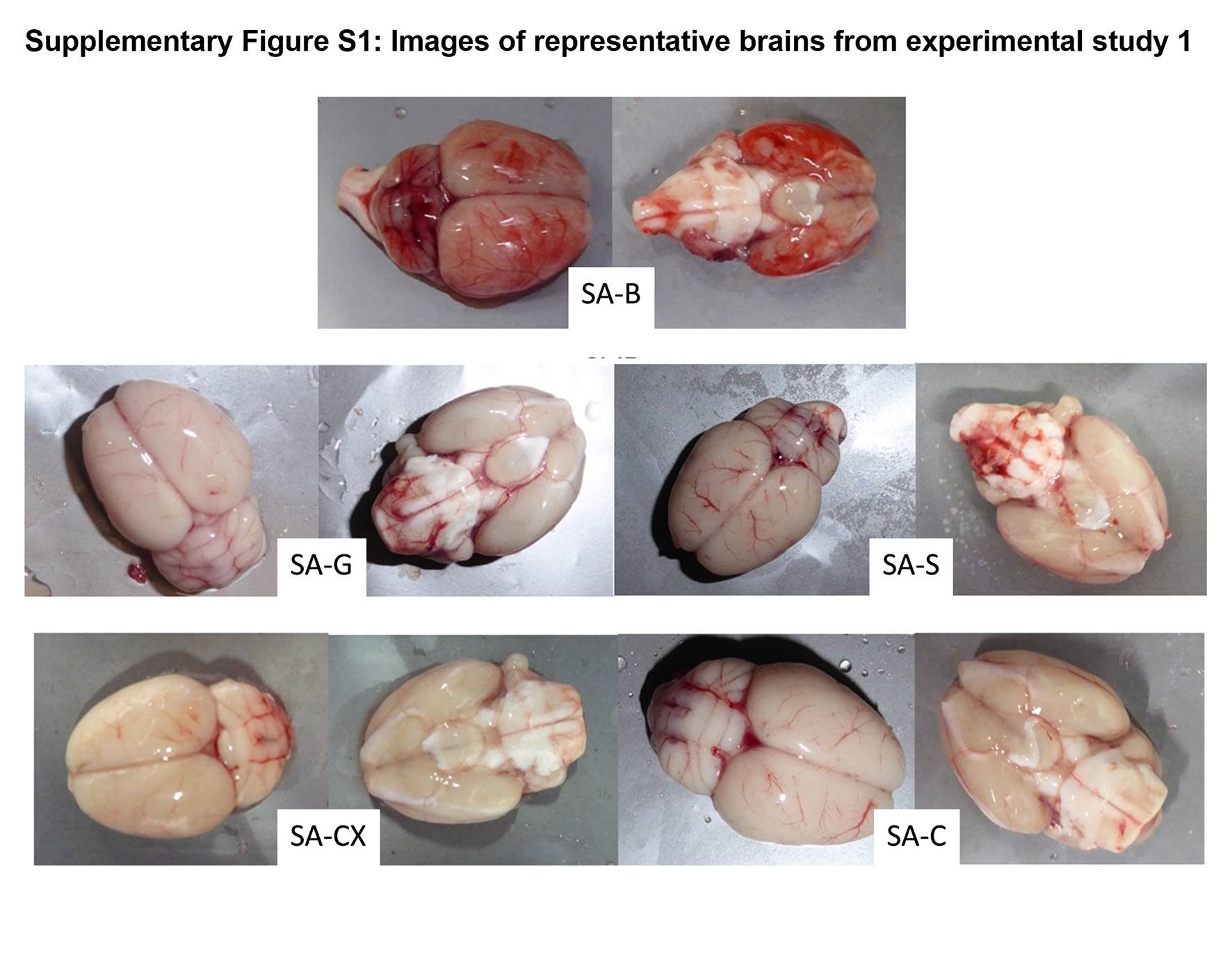

Supplement: Supplementary file 3 [file Image_1.TIF]

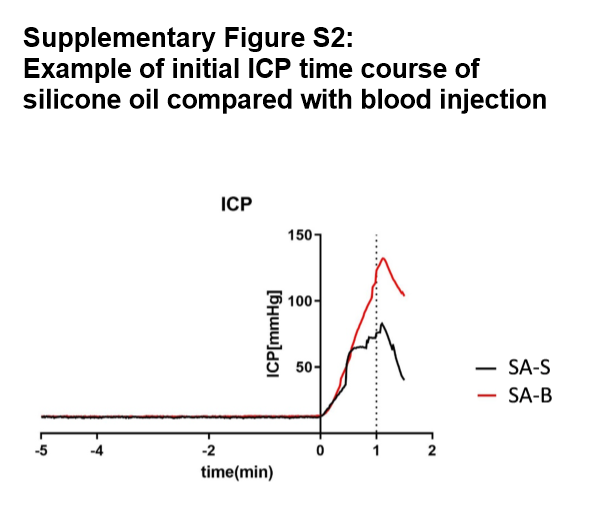

Supplement: Supplementary file 4 [file Image_2.TIF]

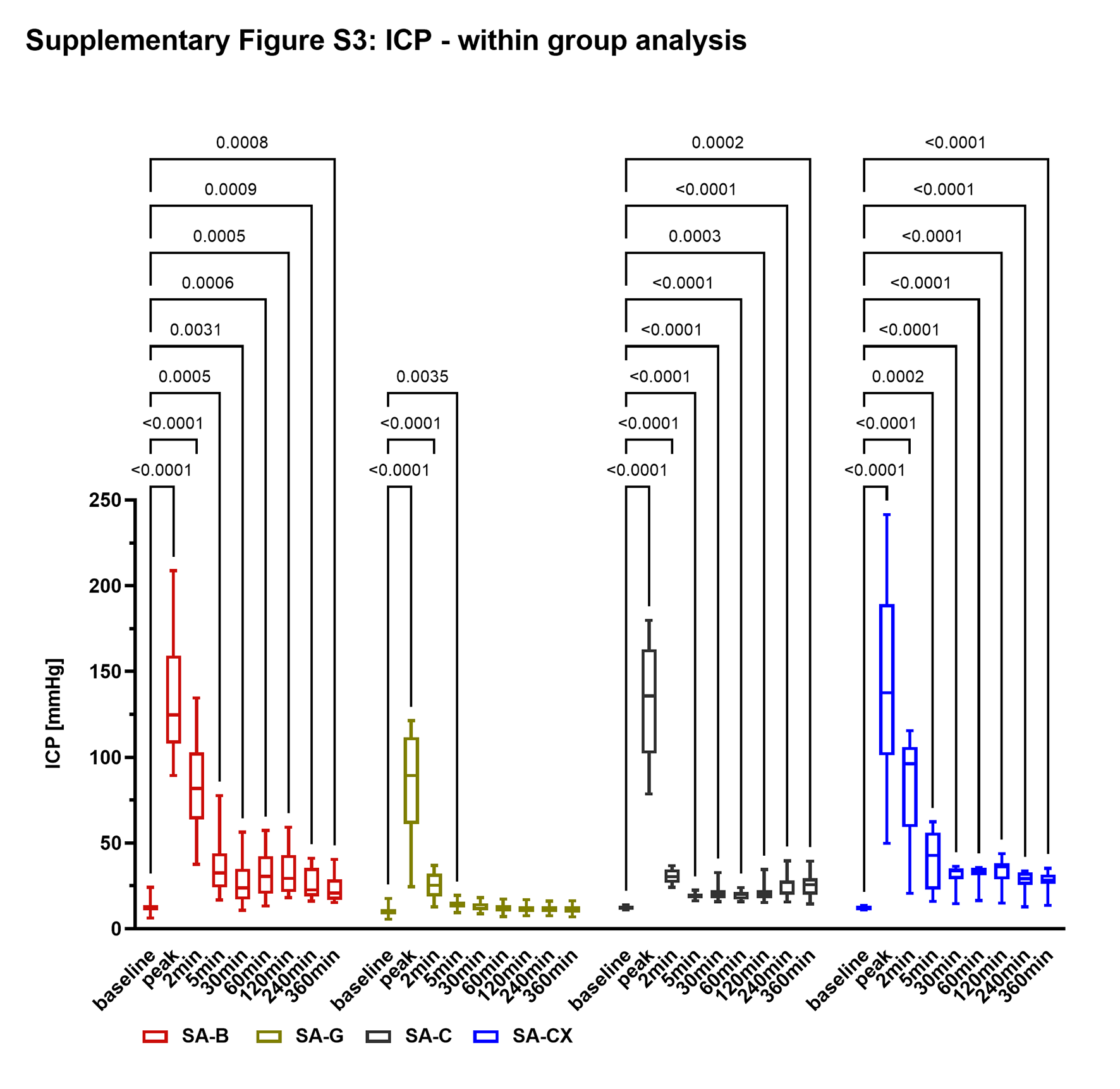

Supplement: Supplementary file 5 [file Image_3.TIF]

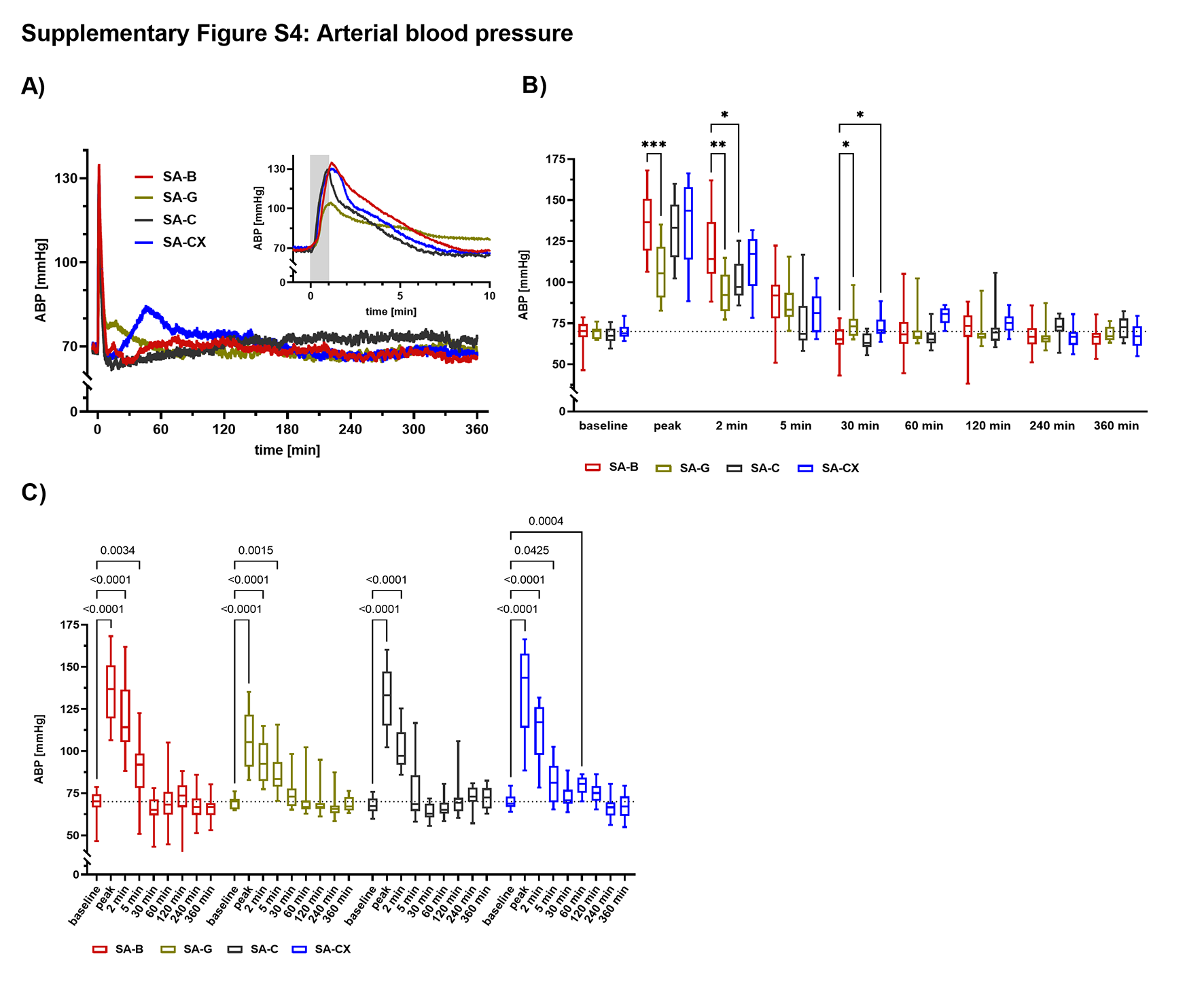

Supplement: Supplementary file 6 [file Image_4.TIF]

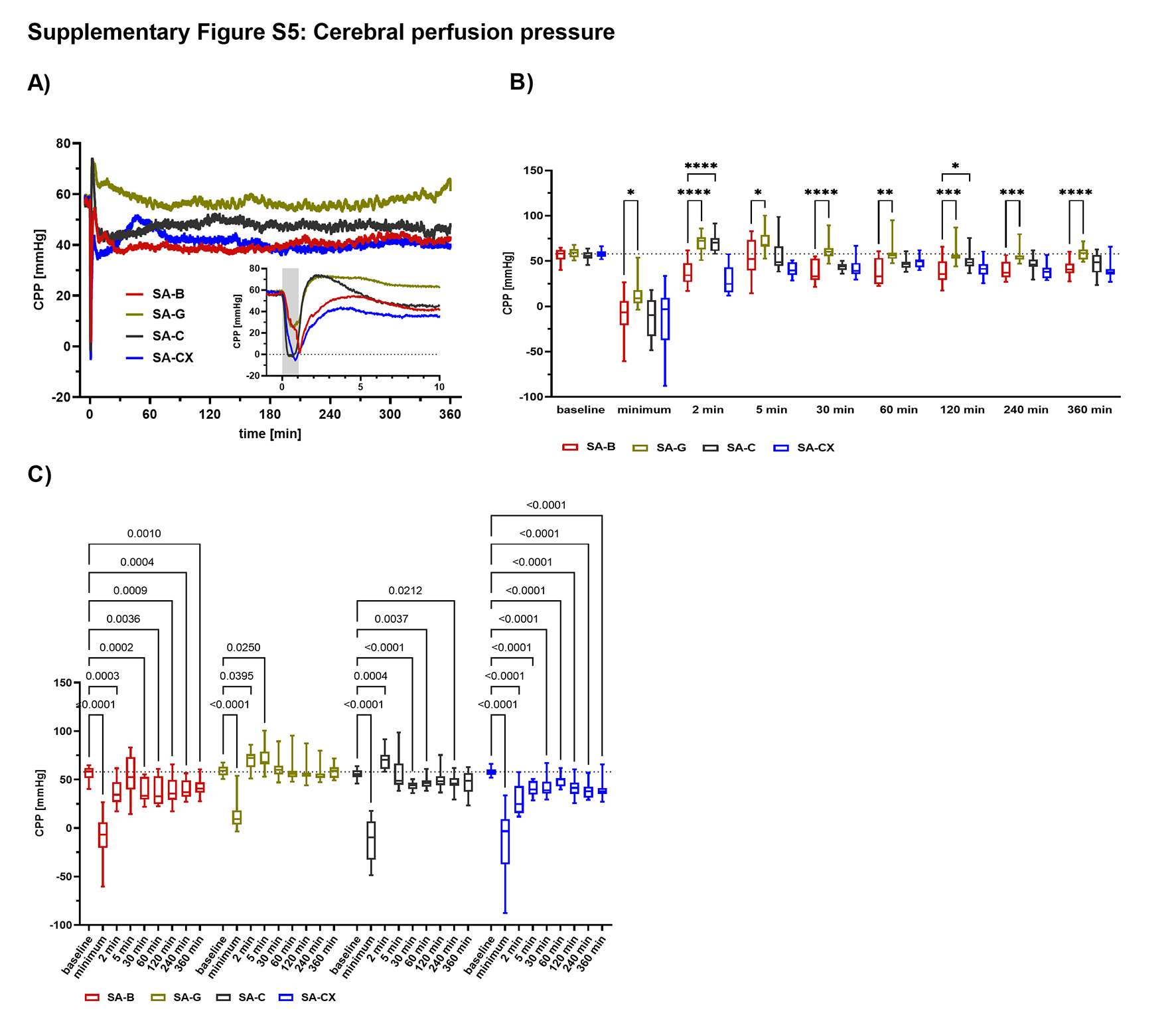

Supplement: Supplementary file 7 [file Image_5.TIF]

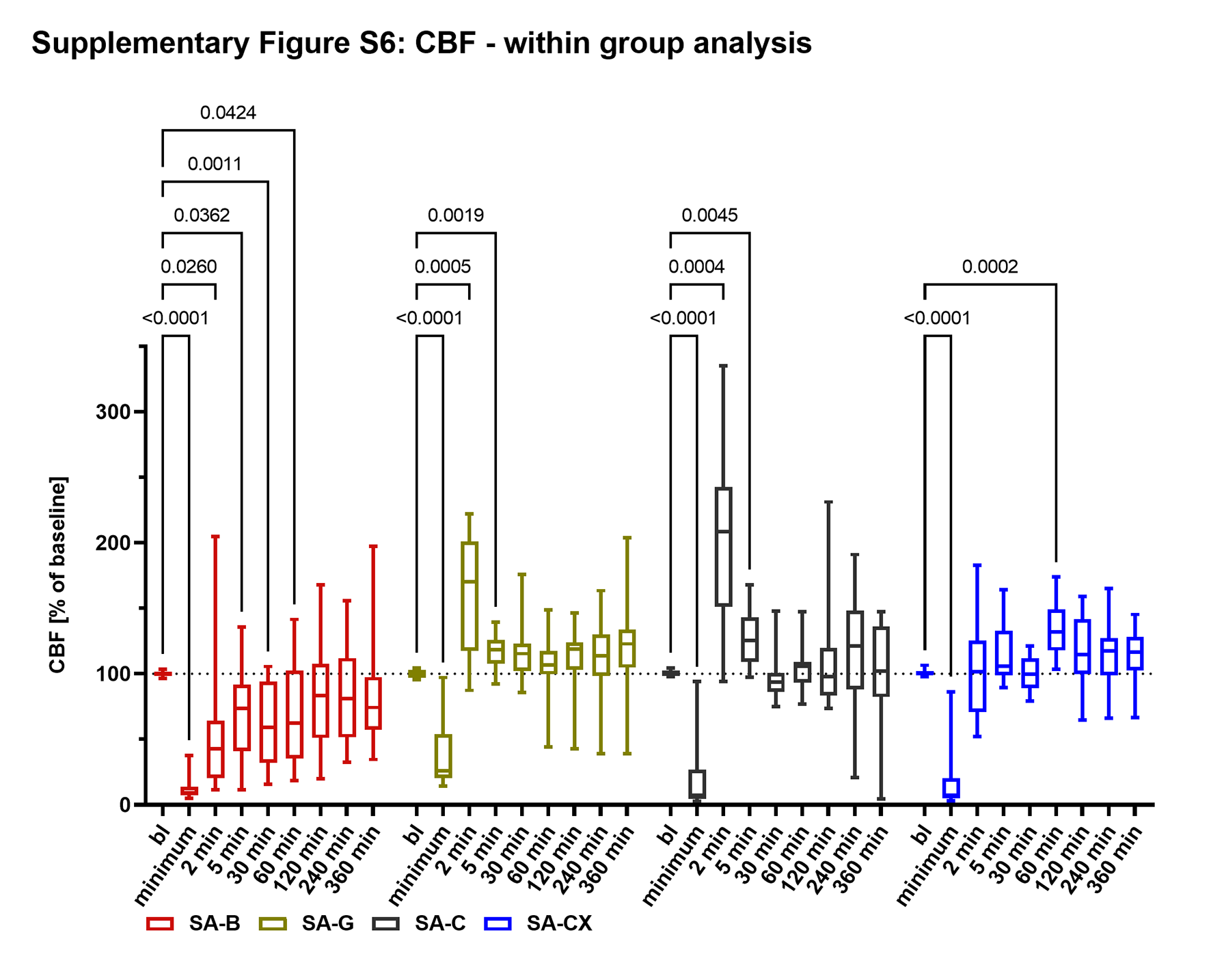

Supplement: Supplementary file 8 [file Image_6.TIF]

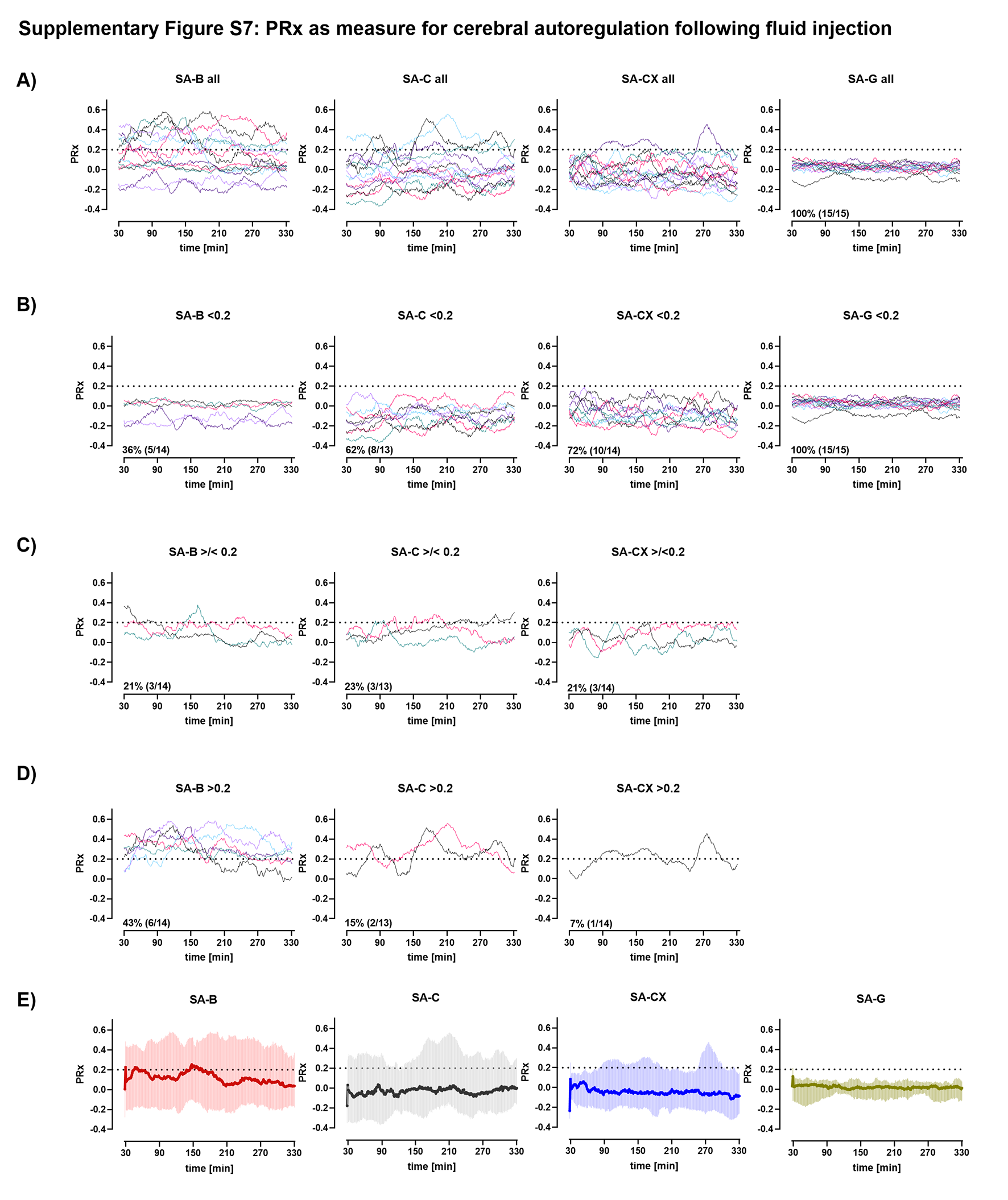

Supplement: Supplementary file 9 [file Image_7.TIF]
